# Supplementary material for: Association between ethnic background and COVID-19 morbidity, mortality and vaccination in England: a multistate cohort analysis using the UK Biobank
Source: BMJ Open. 2023 Sep 21;13(9):e074367. doi: 10.1136/bmjopen-2023-074367 (PMC10514643; doi:10.1136/bmjopen-2023-074367)
Supplement: Supplementary data [file bmjopen-2023-074367supp001.pdf]

Supplementary Figure 1. Radar charts for socio-economic deprivation quintile population distribution, stratified by ethnicity. The distribution of each ethnic group is shown by the coloured pentagons, in comparison to the whole cohort distribution in dark grey beneath.

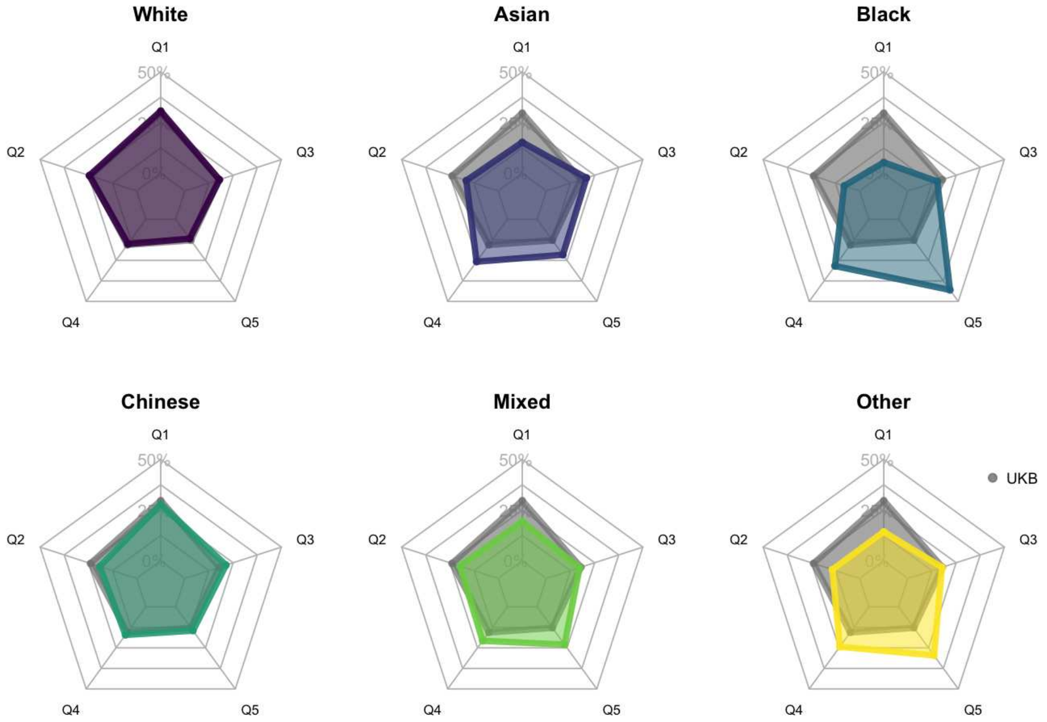

Supplementary Table 1. COVID outcome event numbers. Stratified by ethnicity, socioeconomic deprivation quintile, sex and age. All percentages (in brackets) are calculated as the fraction of individuals who underwent a particular transition from the total who were in the states preceding it (and thus susceptible to transitioning). Age is divided into those younger and older than the median age 70.8.

	Total	UKB-Negative	Negative-Positive	UKB-Positive	Positive-Hospital	Positive-Death	UKB-Hospital	Hospital-Death
All (%)	405541	111614 (27.5)	3285 (2.9)	18172 (4.5)	1490 (6.9)	129 (0.6)	2322 (0.6)	662 (17.4)
**Ethnicity**								
Asian (%)	8791	2132 (24.3)	100 (4.7)	714 (8.1)	68 (8.4)	4 (0.5)	75 (0.9)	25 (17.5)
Black (%)	7275	1883 (25.9)	84 (4.5)	450 (6.2)	52 (9.7)	3 (0.6)	82 (1.1)	19 (14.2)
Chinese (%)	1324	238 (18)	4 (1.7)	31 (2.3)	3 (8.6)	0 (0)	7 (0.5)	2 (20)
Mixed (%)	2537	688 (27.1)	25 (3.6)	146 (5.8)	12 (7)	0 (0)	12 (0.5)	4 (16.7)
Other (%)	3964	1068 (26.9)	53 (5)	226 (5.7)	18 (6.5)	2 (0.7)	28 (0.7)	4 (8.7)
White (%)	381650	105605 (27.7)	3019 (2.9)	16605 (4.4)	1337 (6.8)	120 (0.6)	2118 (0.6)	608 (17.6)
**Socio-economic deprivation**								
Q1 (%)	120443	34121 (28.3)	790 (2.3)	4112 (3.4)	290 (5.9)	34 (0.7)	478 (0.4)	127 (16.5)
Q2 (%)	97002	26912 (27.7)	741 (2.8)	4006 (4.1)	258 (5.4)	20 (0.4)	431 (0.4)	95 (13.8)
Q3 (%)	72708	19607 (27)	585 (3)	3454 (4.8)	286 (7.1)	18 (0.4)	391 (0.5)	132 (19.5)
Q4 (%)	63404	17235 (27.2)	615 (3.6)	3363 (5.3)	294 (7.4)	30 (0.8)	461 (0.7)	138 (18.3)
Q5 (%)	51984	13739 (26.4)	554 (4)	3237 (6.2)	362 (9.5)	27 (0.7)	561 (1.1)	170 (18.4)
**Sex**								

		Total	UKB-Negative	Negative-Positive	UKB-Positive	Positive-Hospital	Positive-Death	UKB-Hospital	Hospital-Death
	Male (%)	181413	51172 (28.2)	1512 (3)	8365 (4.6)	829 (8.4)	72 (0.7)	1281 (0.7)	429 (20.3)
	Female (%)	224128	60442 (27)	1773 (2.9)	9807 (4.4)	661 (5.7)	57 (0.5)	1041 (0.5)	233 (13.7)
**Age**									
	Younger than 70 (%)	201131	51280 (12.6)	1970 (1.8)	12598 (3.1)	729 (3.4)	16 (0.1)	725 (0.2)	129 (3.4)
	Older than 70 (%)	204410	60334 (14.9)	1315 (1.2)	5574 (1.4)	761 (3.5)	113 (0.5)	1597 (0.4)	533 (14)

Supplementary Table 2. Hazard ratio coefficients stratified by ethnicity, at different levels of confounding. Fully adjusted for age, sex, socioeconomic deprivation and comorbidities.

	UKB-Negative	Negative-Positive	UKB-Positive	Positive-Hospital	UKB-Hospital	Hospital-Death
**Asian vs White**						
Unadjusted	0.9 [0.86,0.93]	1.54 [1.26,1.88]	1.91 [1.77,2.06]	1.05 [0.82,1.34]	1.56 [1.24,1.97]	0.93 [0.62,1.39]
Age-sex adjusted	0.94 [0.9,0.98]	1.3 [1.07,1.59]	1.55 [1.44,1.67]	1.26 [0.99,1.61]	1.89 [1.5,2.39]	1.34 [0.9,2.01]
Fully adjusted	0.92 [0.88,0.96]	1.21 [0.99,1.48]	1.4 [1.3,1.51]	0.93 [0.72,1.19]	1.32 [1.04,1.67]	1.23 [0.81,1.85]
**Black vs White**						
Unadjusted	0.95 [0.91,1]	1.48 [1.2,1.84]	1.44 [1.31,1.58]	1.44 [1.09,1.9]	2 [1.6,2.51]	0.82 [0.52,1.3]
Age-sex adjusted	1.02 [0.98,1.07]	1.17 [0.94,1.45]	1.11 [1.01,1.22]	1.96 [1.48,2.59]	2.79 [2.22,3.49]	1.25 [0.79,1.98]
Fully adjusted	1 [0.96,1.05]	1.03 [0.83,1.29]	0.94 [0.85,1.03]	1.51 [1.14,2.01]	1.54 [1.22,1.94]	1.18 [0.74,1.88]
**Chinese vs White**						
Unadjusted	0.6 [0.53,0.69]	0.61 [0.23,1.62]	0.5 [0.35,0.71]	1.28 [0.41,3.99]	0.91 [0.43,1.91]	0.77 [0.19,3.1]
Age-sex adjusted	0.64 [0.57,0.73]	0.51 [0.19,1.36]	0.41 [0.29,0.58]	1.9 [0.61,5.92]	1.24 [0.59,2.61]	1.1 [0.27,4.4]
Fully adjusted	0.67 [0.59,0.76]	0.5 [0.19,1.33]	0.41 [0.29,0.58]	1.78 [0.57,5.56]	1.32 [0.63,2.77]	1.21 [0.3,4.87]
**Mixed vs White**						
Unadjusted	0.99 [0.92,1.06]	1.29 [0.87,1.91]	1.33 [1.13,1.57]	1 [0.57,1.76]	0.87 [0.49,1.53]	0.85 [0.32,2.27]

	UKB-Negative	Negative-Positive	UKB-Positive	Positive-Hospital	UKB-Hospital	Hospital-Death
Age-sex adjusted	1.07 [0.99,1.15]	1 [0.68,1.49]	1.02 [0.87,1.2]	1.41 [0.8,2.5]	1.24 [0.7,2.18]	1.03 [0.39,2.77]
Fully adjusted	1.05 [0.97,1.13]	0.96 [0.65,1.42]	0.95 [0.81,1.12]	1.22 [0.69,2.16]	0.87 [0.49,1.54]	0.83 [0.31,2.24]
**Other vs White**						
Unadjusted	0.98 [0.93,1.04]	1.76 [1.34,2.31]	1.32 [1.16,1.5]	0.84 [0.52,1.33]	1.3 [0.89,1.88]	0.51 [0.19,1.36]
Age-sex adjusted	1.04 [0.98,1.11]	1.41 [1.07,1.85]	1.06 [0.93,1.21]	1.08 [0.68,1.72]	1.72 [1.18,2.49]	0.76 [0.28,2.02]
Fully adjusted	1.04 [0.98,1.11]	1.27 [0.96,1.67]	0.95 [0.83,1.09]	0.94 [0.59,1.51]	1.22 [0.84,1.78]	0.7 [0.26,1.89]

[a] Fully adjusted for age, sex, socio-economic deprivation and comorbidities.

[b] [] <- 95% Confidence intervals
