# Supplementary material for: Association between ethnic background and COVID-19 morbidity, mortality and vaccination in England: a multistate cohort analysis using the UK Biobank
Source: BMJ Open. 2023 Sep 21;13(9):e074367. doi: 10.1136/bmjopen-2023-074367 (PMC10514643; doi:10.1136/bmjopen-2023-074367)
Supplement: Author's manuscript [file bmjopen-2023-074367.draft_revisions.pdf]

BMJ Open

BMJ Open is committed to open peer review. As part of this commitment we make the peer review history of every article we publish publicly available.

When an article is published we post the peer reviewers' comments and the authors' responses online. We also post the versions of the paper that were used during peer review. These are the versions that the peer review comments apply to.

The versions of the paper that follow are the versions that were submitted during the peer review process. They are not the versions of record or the final published versions. They should not be cited or distributed as the published version of this manuscript.

BMJ Open is an open access journal and the full, final, typeset and author-corrected version of record of the manuscript is available on our site with no access controls, subscription charges or pay-per-view fees (http://bmjopen.bmj.com).

If you have any questions on BMJ Open's open peer review process please email info.bmjopen@bmj.com

BMJ Open

The association between ethnic background and COVID-19 morbidity, mortality and vaccination in England: a multi-state cohort analysis using the UK Biobank

Journal:	*BMJ Open*
Manuscript ID	bmjopen-2023-074367
Article Type:	Original research
Date Submitted by the Author:	11-Apr-2023
Complete List of Authors:	Urdiales, Tomás; Universitat Politècnica de Catalunya, Physics Department; Royal Institute of Technology, Department of Energy Technolgy<br>Dernie, Francesco; University of Oxford,<br>Català, Martí; University of Oxford, Pharmaco- and Device Epidemiology, Centre for Statistics in Medicine, Nuffield Department of Orthopaedics, Rheumatology and Musculoskeletal Sciences<br>Prats, Clara; Universitat Politècnica de Catalunya, Physics Department<br>Prats-Uribe, Albert; University of Oxford Centre for Statistics in Medicine, Nuffield Department of Orthopaedics Rheumatology and Musculoskeletal Science<br>Prieto-Alhambra, Daniel; University of Oxford Centre for Statistics in Medicine, NDORMS
Keywords:	COVID-19, Public health < INFECTIOUS DISEASES, Epidemiology < INFECTIOUS DISEASES

SCHOLARONE™
Manuscripts

I, the Submitting Author has the right to grant and does grant on behalf of all authors of the Work (as defined in the below author licence), an exclusive licence and/or a non-exclusive licence for contributions from authors who are: i) UK Crown employees; ii) where BMJ has agreed a CC-BY licence shall apply, and/or iii) in accordance with the terms applicable for US Federal Government officers or employees acting as part of their official duties; on a worldwide, perpetual, irrevocable, royalty-free basis to BMJ Publishing Group Ltd ("BMJ") its licensees and where the relevant Journal is co-owned by BMJ to the co-owners of the Journal, to publish the Work in this journal and any other BMJ products and to exploit all rights, as set out in our licence.

The Submitting Author accepts and understands that any supply made under these terms is made by BMJ to the Submitting Author unless you are acting as an employee on behalf of your employer or a postgraduate student of an affiliated institution which is paying any applicable article publishing charge ("APC") for Open Access articles. Where the Submitting Author wishes to make the Work available on an Open Access basis (and intends to pay the relevant APC), the terms of reuse of such Open Access shall be governed by a Creative Commons licence – details of these licences and which Creative Commons licence will apply to this Work are set out in our licence referred to above.

Other than as permitted in any relevant BMJ Author's Self Archiving Policies, I confirm this Work has not been accepted for publication elsewhere, is not being considered for publication elsewhere and does not duplicate material already published. I confirm all authors consent to publication of this Work and authorise the granting of this licence.

For peer review only

The association between ethnic background and COVID-19 morbidity, mortality and vaccination in England: a multi-state cohort analysis using the UK Biobank

AUTHORS: Tomás Urdiales[1,2], Francesco Dernie[3], Martí Català[4], Albert Prats-Uribe[4]*, Clara Prats[1], and Daniel Prieto-Alhambra[4,5]**

*Correspondence to: Albert Prats-Uribe (albert.prats-uribe@ndorms.ox.ac.uk)
** Senior author

1. Physics Department, Universitat Politècnica de Catalunya, Barcelona, Spain.

2. Department of Energy Technology, Royal Institute of Technology (KTH), Stockholm, Sweden.

3. University of Oxford Medical School, Medical Sciences Divisional Office, University of Oxford, Oxford, OX3 9DU, UK.

4. Pharmaco- and Device Epidemiology, Centre for Statistics in Medicine, Nuffield Department of Orthopaedics, Rheumatology and Musculoskeletal Sciences, University of Oxford, Oxford, UK.

5. Department of Medical Informatics, Erasmus Medical Centre University, Rotterdam, Netherlands

For peer review only

Abstract

Objectives: Despite growing evidence suggesting increased COVID-19 mortality among people from ethnic minorities, little is known about milder forms of SARS-CoV-2 infection. We sought to explore the association between ethnic background and the probability of testing, testing positive, hospitalisation, COVID-19 mortality, and vaccination uptake.

Design: A multi-state cohort analysis. Participants were followed between 8th April 2020 to 30th September 2021.

Setting: The UK Biobank, which stores medical data on around half a million people who were recruited between 2006-2010.

Participants: 405,541 subjects were eligible for analysis, limited to UK Biobank participants living in England. 23,891 (6%) of participants were non-white.

Primary and secondary outcome measures: The associations between ethnic background and testing, testing positive, hospitalisation and COVID-19 mortality were studied using multi-state survival analyses. The association with single and double-dose vaccination was also modelled. Multi-state models adjusted for age, sex, and socio-economic deprivation were fitted to estimate adjusted Hazard Ratios (aHR) for each of the multi-state transitions

Results: 18,172 (4.5%) individuals tested positive, 3,285 (0.8%) tested negative and then positive, 1,490 (6.9% of those tested positive) were hospitalised, and 129 (0.6%) tested positive at the moment of hospital admission (i.e., direct hospitalisation). Finally, 662 (17.4%) died after admission. Compared to White participants, Asian participants had an increased risk of negative to positive transition (aHR 1.24 [1.02-1.52]), testing positive (1.44 [1.33-1.55]) and direct hospitalisation (1.61 [1.28-2.03]). Black participants had an increased risk of hospitalisation following a positive test (1.71 [1.29-2.27]) and direct hospitalisation (1.90 [1.51-2.39]). Although not the case for Asians (aHR 1.00 [0.98-1.02]), Black participants had a reduced vaccination probability (0.63 [0.62-0.65]). In contrast, Chinese participants had a reduced risk of testing negative (aHR 0.64 [0.57-0.73]), of testing positive (0.40 [0.28-0.57]), and of vaccination (0.78 [0.74-0.83]).

Conclusions: We identified inequities in testing, vaccination and COVID-19 outcomes according to ethnicity in England. Compared to Whites, Asian participants had increased risks of infection and admission, and Black participants had almost double hospitalisation risk, and a 40% lower vaccine uptake.

For peer review only

Strengths and limitations of this study

- This study uses a large cohort of adults, allowing for high powered assessment of associations between baseline characteristics and COVID outcomes
- Key confounders are adjusted for in our statistical analysis
- Our analyses provide new insights into the link between ethnicity and a range of COVID outcomes
- The UK Biobank cohort is not optimally representative, limiting extrapolation of findings to the wider population

For peer review only

Introduction

Since the first cases of human infection with SARS-CoV-2 were reported in Wuhan, China in late 2019, as of the 15th of March 2022 the COVID-19 pandemic has seen more than 450 million confirmed cases and 6 million confirmed deaths worldwide, causing unprecedented economic and social disruption.[1] Despite its global reach, it has become increasingly clear that the risks of initial infection, death and long-term consequences from COVID-19 are not distributed equally in society, including between different ethnic groups.[2]

In the early phase of the pandemic, initial studies sought to characterise factors associated with COVID infection in patients admitted to hospital,[3,4] as only these patients received formal COVID tests at the time. However, by focussing on this limited patient group, these studies had intrinsic biases which limited their generalisability to the wider population.[5]

To address these concerns, studies have sought to study COVID-19 infection risk in larger population cohorts, such as the established UK Biobank project.[6] Early in the pandemic, COVID-19 test results for UK Biobank participants were linked to their biobank data, allowing participants' COVID status to be followed.[7] These studies have identified several factors, including age, sex, comorbidities, ethnicity and socioeconomic status as significant determinants of COVID-19 infection, hospitalisation, and mortality.[8–23] Specifically, studies have suggested that Black and Asian people are at increased risk of testing positive, being hospitalised and dying from COVID-19, compared to White people. [9–12,16,18–21,23]

However, these studies were also prone to limitations. Firstly, many gathered information from the UK Biobank only during the first wave of COVID-19 (February-May 2020), meaning there were relatively few positive cases to compare to the rest of the cohort, and wider population testing (so-called 'pillar 2' testing) only became available in the UK in early April 2020. Secondly, many only dealt with individual outcome measures – either testing positive, being hospitalised, or death – meaning that the rest of the individual's journey through COVID-19 was not comprehensively assessed. This lack of detail may mask some differences between groups, thus limiting the implementation of findings concerning the role of ethnicity into public health policy in both the current and future pandemics.

This paper seeks to address several of these issues to delineate the influence of ethnicity on a variety of COVID-19 outcomes. We use UK Biobank data from the start of pillar 2 testing (April 2020) until September 2021, allowing an up-to-date comparison of a higher number of positive cases, across multiple waves of the pandemic. We analyse this data using a multistate model to be able to study participants' transitions between different 'COVID states'. given that the outcome of interest was just the first transition into any of the stage of a journey through COVID-19. .

For peer review only

Methods

Study design, setting and data sources

We conducted a cohort study using the UK Biobank cohort[5], a large, non-commercial, long-term biobank project in the United Kingdom which stores medical data on around half a million people who were recruited between 2006-2010. All participants were aged between 40-69 years at the time of their registration with the project and will remain in follow-up for a minimum of 30 years. All participants provided demographic and lifestyle information, as well as blood, urine, and saliva samples. COVID-19 testing data was provided by the UK Biobank through dynamic linkage with Public Health England's Second Generation Surveillance System.[7] This data is only available for England and has 108 laboratories reporting positive test, and 101 reporting negative tests too. Data on deaths was taken from the Office of National Statistics, which the UK Biobank updates regularly for their participants., and it is fully linked for England. [24]

We followed participants in this programme from the 8th April 2020 to 30th September 2021, which is representative of the start of wider population testing (pillar 2) for COVID-19 in the UK to the latest available data entry point common to all different data sources (testing, hospitalisation, mortality records) at the time of analysis and writing. We limited our analysis to participants in England, given that England has a different multiple economic deprivation index to the other constituent countries of the UK, and testing data was only available for England.

Data from the UK Biobank was combined with National Health Service (NHS) Hospital Episode Statistics (HES) Admitted Patient Care (APC) data,[25] a programme which stores data on all hospital admissions, outpatient appointments and attendances, to build a comprehensive database that gathered information on COVID-19 testing, hospitalisation and mortality, as well as key variables including age, sex, ethnicity, and socioeconomic status for the entire cohort.

Multistate framework

A multi-state model [26] served as the framework for analysis, which has been used previously to describe risks of COVID-19 diagnosis, hospitalisation and death elsewhere.[27] These models allow the characterisation and analysis of individual transitions between different 'COVID states'. In this study, all participants began as part of the UK Biobank Cohort (UKB), who had never been tested for COVID-19. They could then progress to one of three separate states: testing negative, testing positive, or direct hospitalisation (where testing positive for COVID-19 occurred on the same day as hospitalisation). Addition of a fifth health state of 'death' allowed for seven separate possible transitions: UKB to negative, UKB to positive, UKB to hospital, negative to positive, positive to hospital, positive to death (where hospitalisation occurred on the same day as death, or the patient was never hospitalised), and hospital to death (Figure 1). Only the first negative test and subsequent positive test, or the first

For peer review only

positive test, were counted, and each transition is unidirectional, so once an individual had a positive test, any subsequent negative tests are discounted.

Participants and study size

We initially included all participants of the UK Biobank programme. We excluded participants if they live outside of England, had died prior to the start of follow-up (8th April 2020), had requested that their data not be used for research, or had either missing data on ethnic background or had answered 'Do not know' or 'Prefer not to say' to questions asking about ethnic background.

Variables

The main 'exposure' of interest was ethnicity. Ethnicity was categorised into six groups, in line with the ethnic groupings specified by the UK Biobank at the time of recruitment to their programme. These are: Asian, Black, Chinese, Mixed, White and 'other'. Further detail on the ethnicities comprising each group can be found in the UKBiobank showcase (https://biobank.ndph.ox.ac.uk/showcase/field.cgi?id=21000).Language used in the reporting and discussion of ethnicity in the text is in line with published recommendations.[28] The main covariates for multivariable adjustment were sex, age and socioeconomic deprivation (Index of Multiple Deprivation quintiles). and comorbidities The Index of Multiple Deprivation combines information from seven unique domains of deprivation, to provide an overall relative measure of deprivation for 32,844 neighbourhoods in England (Lower-layer Super Output Areas). [29] Comorbidities were assessed using both hospitalisation data (HES) and Primary Care at the time of start of follow-up (8th April 2020). We used ICD-10 for HES,[30] and SNOMED and READ codes for primary care used previously,[31] using the categories of the Charlson comorbidity index.

Outcomes

The outcomes of interest were results of COVID tests, hospitalisation with COVID-19 and COVID-19-related death, gathered using the data sources discussed above. We deemed as COVID-19 related death or hospitalisation with COVID those which occurred within 28 days of a positive SARS-CoV-2 test. COVID-19 was not required to be the primary diagnosis or reason for hospitalisation. Data on whether tests were positive or not was obtained from Public Health England as described elsewhere.[7]

Test Count: Another outcome of interest was the test count, defined as the number of tests taken for each patient since the start of the follow-up period until the end of it.

Receiving a COVID-19 vaccine was defined as having a code READ code "Y29e7" or SNOMED CT "1324681000000101" for the first dose and "Y29e8" or "1324691000000104" for the second dose on the Primary Care data.

For peer review only

Statistical methods

Baseline characteristics are presented as n (%) or median (IQR) overall and for each ethnicity category. Incidence of outcomes are shown as % of people who transition from one state to another during the whole study period. A Cox proportional hazards model was used to determine hazard ratios for each group undergoing each transition in the multi-state model, adjusted for the confounders outlined above. A 'clock forward' approach, modelling all transitions in the same timescale (time since entering the study), for time-to-events was used. The proportional hazards assumption was checked using the Kaplan-Meier estimator.[32]

Quasi-Poisson (to account for overdispersion) regressions were used to analyse the number of tests undertaken by each participant. It was further stratified by ethnic background.

For the analyses of vaccines, a Cox proportional hazards models was again used to determine hazard ratios for each variable and transition: from unvaccinated to first dose, and from first dose to second dose.

All statistical analyses were carried out using R: the *tidyverse* collection of packages for data curation,[33] the *mstate* and *survival* packages for multistate modelling and statistical regressions,[34,35] and *kable* and *kableExtra* for graphical representation. [36]

Hazard ratios for the Positive to Death transition are not shown, as most groups had no individuals undergoing this transition, causing confidence intervals which were too wide to provide real information about relative risk.

Patient and public involvement

No funding was available for patient or public involvement in this project. No patients were involved in setting the research question or the outcome measures. Patients were not invited to comment on the study design, not consulted to develop patient relevant outcomes or interpret the results, and not invited to contribute to the writing or editing of this document for readability or accuracy.

Results

Baseline characteristics

After initial inclusion of all participants of the UK Biobank programme (n=502,510), a total of 405,541 (80.7%) participants were included in the final analysis (Figure 2). The baseline characteristics of the final population are summarised in Table 1. Our cohort was predominantly White with 381,650 (94.1%), and the majority were female (224,128 - 55.3%). The median age was 70.5 years old, but the median age of White participants was around five years older than participants of other ethnic groups. The upper quintiles of socio-economic status were overrepresented in the cohort overall (29.7% in Q1 vs 12.8% in Q5), and there was clear disparity in the distribution of participants between different quintiles based on ethnicity (Supplementary Figure 1).

For peer review only

Table 1. Cohort population baseline characteristics. Data is stratified by ethnicity and displays sex, age, socio-economic status and comorbidities. Age is presented by group median age alongside interquartile range. All percentages (in brackets) are calculated with respect to group size *n*.

	All	Asian	Black	Chinese	Mixed	Other	White
**n**	405541	8791	7275	1324	2537	3964	381650
**Sex=Male (%)**	181413 (44.7)	4642 (52.8)	3017 (41.5)	468 (35.3)	925 (36.5)	1685 (42.5)	170676 (44.7)
**Age (median [IQR])**	70.5 [62.8,75.8]	65.2 [58.7,72.5]	63 [57.9,70]	65.1 [58.9,70.9]	62.8 [57.6,70.4]	64.6 [58.3,71.3]	70.8 [63.2,76.1]
**Socio-economic deprivation**							
**Q1 (%)**	120443 (29.7)	1345 (15.3)	390 (5.4)	370 (27.9)	492 (19.4)	567 (14.3)	117279 (30.7)
**Q2 (%)**	97002 (23.9)	1452 (16.5)	584 (8)	253 (19.1)	502 (19.8)	559 (14.1)	93652 (24.5)
**Q3 (%)**	72708 (17.9)	1825 (20.8)	1106 (15.2)	282 (21.3)	436 (17.2)	703 (17.7)	68356 (17.9)
**Q4 (%)**	63404 (15.6)	2268 (25.8)	2068 (28.4)	227 (17.1)	524 (20.7)	962 (24.3)	57355 (15)
**Q5 (%)**	51984 (12.8)	1901 (21.6)	3127 (43)	192 (14.5)	583 (23)	1173 (29.6)	45008 (11.8)
**Comorbidities**							
**Myocardial infarction (%)**	16389 (4)	580 (6.6)	152 (2.1)	15 (1.1)	75 (3)	137 (3.5)	15430 (4)
**Congestive heart failure (%)**	11282 (2.8)	267 (3)	178 (2.4)	7 (0.5)	36 (1.4)	89 (2.2)	10705 (2.8)
**Peripheral vascular disease (%)**	11643 (2.9)	242 (2.8)	140 (1.9)	17 (1.3)	54 (2.1)	75 (1.9)	11115 (2.9)
**Cerebrovascular disease (%)**	18968 (4.7)	347 (3.9)	324 (4.5)	26 (2)	92 (3.6)	157 (4)	18022 (4.7)
**Dementia (%)**	4299 (1.1)	80 (0.9)	87 (1.2)	5 (0.4)	16 (0.6)	24 (0.6)	4087 (1.1)
**Chronic pulmonary disease (%)**	77899 (19.2)	1761 (20)	1315 (18.1)	183 (13.8)	536 (21.1)	719 (18.1)	73385 (19.2)
**Rheumatologic disease (%)**	15635 (3.9)	373 (4.2)	305 (4.2)	24 (1.8)	92 (3.6)	128 (3.2)	14713 (3.9)
**Peptic ulcer disease (%)**	15830 (3.9)	381 (4.3)	334 (4.6)	60 (4.5)	94 (3.7)	191 (4.8)	14770 (3.9)
**Mild liver disease (%)**	9890 (2.4)	224 (2.5)	200 (2.7)	50 (3.8)	69 (2.7)	139 (3.5)	9208 (2.4)
**Diabetes (%)**	42222 (10.4)	2687 (30.6)	1693 (23.3)	161 (12.2)	301 (11.9)	817 (20.6)	36563 (9.6)
**Hemiplegia (%)**	3278 (0.8)	80 (0.9)	95 (1.3)	6 (0.5)	20 (0.8)	32 (0.8)	3045 (0.8)

For peer review only

	All	Asian	Black	Chinese	Mixed	Other	White
**Moderate or severe renal disease (%)**	27065 (6.7)	631 (7.2)	750 (10.3)	47 (3.5)	155 (6.1)	246 (6.2)	25236 (6.6)
**Cancer (%)**	55122 (13.6)	665 (7.6)	703 (9.7)	106 (8)	255 (10.1)	365 (9.2)	53028 (13.9)
**AIDS (%)**	533 (0.1)	9 (0.1)	41 (0.6)	3 (0.2)	5 (0.2)	17 (0.4)	458 (0.1)

Occurrence of COVID-19 outcomes

Of the entire cohort, 18172 (4.5%) individuals tested positive, and a further 3285 (0.8%) tested positive following a previous negative result. 1490 individuals (6.9% of those who tested positive) were hospitalised following a positive result, and a further 2322 (0.6%) were directly hospitalised. Of those who were hospitalised, 662 individuals (17.4%) subsequently died within 28 days of a positive COVID test. Full descriptive statistics of the transitions between each COVID state are shown in Supplementary Table 1, stratified by ethnicity, socio-economic deprivation, sex and age.

Risks of testing positive, hospitalisation and death

Stratification of the cohort by ethnicity revealed differences between different ethnic groups in their risk of transitioning between different COVID states (Figure 3, Supplementary Table 2). Compared to White participants, Asian participants had an increased risk of testing positive (1.40 [1.30-1.51]) and direct hospitalisation (1.32 [1.04-1.67]), and a reduced risk of testing negative (0.92 [0.88-0.95]). Black participants had an increased risk of hospitalisation following a positive test (1.51 [1.14-2.01]) and direct hospitalisation (1.54 [1.22-1.94]). In contrast, Chinese participants had a reduced risk of testing negative (0.67 [0.59-0.76]) and testing positive (0.41 [0.29-0.58]). Participants with mixed or 'other' ethnicity did not have any notable differences in their risks of transitioning between any states compared with White participants. Once hospitalised, no ethnic group had increased risk of death compared to white participants.

Given the multistate model only accounts for the first positive or negative test an individual takes, Quasi-Poisson regression was used to compare differences in the amount of COVID testing between groups (Table 2). Black (1.15 [1.08-1.22]) and 'other ethnicity' (1.13 [1.03-1.23]) participants had an increased likelihood of testing for COVID-19, whereas in contrast Chinese participants had a reduced likelihood (0.70 [0.58-0.86]).

For peer review only

Table 2. Quasi-Poisson regression coefficients for number of tests taken, stratified by ethnicity, and shown at different levels of confounding. Fully adjusted includes adjustment for age, sex and socio-economic deprivation. Risk ratios are the exponential of the regression estimates, with the corresponding confidence intervals. Quasi-Poisson model results are shown due to overdispersion in the sample.

	**Quasi-Poisson**<br>**Risk ratio [95% CI]**
**Asian vs White**	
Unadjusted	1.10 [1.04,1.17]
Age-sex adjusted	1.14 [1.07,1.22]
Fully adjusted	1.04 [0.97,1.10]
**Black vs White**	
Unadjusted	1.27 [1.19,1.35]
Age-sex adjusted	1.35 [1.26,1.44]
Fully adjusted	1.15 [1.08,1.22]
**Chinese vs White**	
Unadjusted	0.64 [0.52,0.78]
Age-sex adjusted	0.68 [0.55,0.83]
Fully adjusted	0.70 [0.58,0.86]
**Mixed vs White**	
Unadjusted	0.98 [0.87,1.1]
Age-sex adjusted	1.05 [0.93,1.19]

For peer review only

Fully adjusted	0.98 [0.87,1.10]
**Other vs White**	
Unadjusted	1.15 [1.05,1.26]
Age-sex adjusted	1.22 [1.11,1.33]
Fully adjusted	1.13 [1.03,1.23]

[a] Fully adjusted for age, sex and socio-economic deprivation.

[b] [] <- 95% Confidence intervals

In addition, we extracted data concerning COVID-19 vaccine uptake among our cohort, covering both the first and second doses of the vaccine (Table 3). With regards to the first dose, all ethnic groups except Asian were less likely to have been vaccinated at the time of writing, compared to White participants. Asian and mixed ethnicity had similar chances than White participants to receive their second dose of the vaccine, whereas Black and Chinese participants were less likely to.

Table 3. Uptake risk of first and second doses of COVID-19 vaccines, stratified by ethnic background.

	First dose	Second dose
**Asian vs White**		
Unadjusted	0.82 [0.8,0.83]	0.86 [0.84,0.88]
Age-sex adjusted	0.99 [0.96,1.01]	1.08 [1.05,1.10]
Fully adjusted	0.99 [0.97,1.01]	1.09 [1.06,1.11]
**Black vs White**		

For peer review only

Unadjusted	0.52 [0.51,0.54]	0.69 [0.67,0.71]
Age-sex adjusted	0.61 [0.60,0.63]	0.79 [0.77,0.81]
Fully adjusted	0.63 [0.61,0.64]	0.81 [0.78,0.83]
**Chinese vs White**		
Unadjusted	0.65 [0.61,0.69]	0.74 [0.69,0.78]
Age-sex adjusted	0.78 [0.74,0.83]	0.90 [0.85,0.95]
Fully adjusted	0.79 [0.74,0.83]	0.90 [0.85,0.95]
**Mixed vs White**		
Unadjusted	0.66 [0.64,0.69]	0.75 [0.72,0.79]
Age-sex adjusted	0.85 [0.81,0.89]	1.03 [0.99,1.08]
Fully adjusted	0.85 [0.82,0.89]	1.04 [1.00,1.09]
**Other vs White**		
Unadjusted	0.62 [0.60,0.64]	0.8 [0.77,0.82]
Age-sex adjusted	0.74 [0.71,0.76]	1.01 [0.97,1.04]
Fully adjusted	0.76 [0.73,0.79]	1.02 [0.99,1.06]

[a] Fully adjusted for age, sex, socio-economic deprivation and comorbidities.

[b] [] <- 95% Confidence intervals

For peer review only

Discussion

Statement of principle findings

Our study of 405,541 participants of the UK Biobank demonstrates significant differences in risks of various COVID-related outcomes between different ethnic groups. Compared to White participants, we found that Black and Asian participants have clearly increased risks of testing positive for COVID-19 and of being hospitalised for the disease. Whilst our data suggests an increased risk of death following hospitalisation, the high uncertainty for this transition means that higher risk cannot be reliably asserted.

Both Asian and Black participants had a higher likelihood of being tested for COVID-19, whereas Chinese participants were less likely to be, compared to White participants. In terms of vaccine uptake, our study shows White and Asian participants had a higher likelihood of receiving their first vaccine dose compared to other ethnic groups. Compared to White participants, Asian and mixed ethnicity participants were more likely to receive their second vaccine dose, whereas Black and Chinese participants were less likely to.

Strengths and weaknesses of the study

Our study has several strengths. Firstly, it includes a large number of participants from an established database, allowing extraction of both baseline characteristics as well as linked information related to COVID-19 testing and hospitalisation, helping to avoid issues around the self-reporting of COVID-related events and outcomes. Secondly, by choosing a start date for follow-up to coincide with the initiation of wider population testing in the community for COVID-19, we address issues of collider bias intrinsic to similar studies that focussed on the early first wave of the pandemic, when testing was more limited. Thirdly, our follow-up window lasts for 18 months, allowing the inclusion of higher numbers of COVID-19 cases, as well as providing an up-to-date analysis now that the UK has experienced multiple waves of the pandemic.

However, our study also has some weaknesses. Comparative studies have shown that the UK Biobank is not optimally representative of the wider population, with UK Biobank participants tending to be older, more female, healthier, and less socioeconomically deprived, as we show in our description of our cohort's baseline characteristics, which limits the generalisability of our findings. [37] Our approach to confounders helps to address some of these issues. but we still have a very selected population Although we adjust for socio-economic status as a confounder, the deprivation index used is based on territorial units and not on individual characteristics, so it does not contain information pertaining to an individual. For example, we don't measure occupation, an important driver of COVID, and most of our population is in retirement age.

For peer review only

An important consideration is the potential confounding effect that differential vaccine uptake, illustrated in our results above, may have on our primary outcomes in the multi-state model. Given the established effectiveness of vaccines in preventing severe disease, the vaccinated status of individuals in our study may have affected their risk of progressing through our multi-state model. By design, our study could not include individuals who developed COVID-19 but did not test for it, or individuals who died with undiagnosed COVID-19. Also, the number of outcomes other than negative or positive tests in some ethnicity categories is quite low, so those should be interpreted with caution. We did not evaluate interactions that may be relevant, such as gender, age and SES with ethnicity, due to low number of outcomes. We also recognise that the UK Biobank's initial categorisation of ethnicity, used by necessity in this study, differs from the current UK Government Statistical Service ethnicity harmonised standard. Differences in linkage to tests, with some laboratories not reporting negative tests, and availability and differential use and reporting of home testing could also have biased the results.

Strengths and weaknesses in relation to other studies, discussing important differences in results

Our study has several strengths in comparison to previous studies examining COVID outcomes using the UK Biobank. Our longer observation period and use of start dates in conjunction with wider population testing helps address intrinsic biases, and our multistate approach allows a more detailed assessment of different groups risks of specific outcomes than previously reported.[38]

This approach has allowed us to confirm several findings of previous studies, mainly conducted in a timeframe where testing was only performed on hospital, namely that Black and Asian individuals have a higher risk of testing positive for COVID-19 (indicating severe disease as most where hospitalised). [2,11,23,39,40] We found that once more wide availability of tests started, we still find an increased risk of testing positive in hospital for Black and Asian people, and increased risk of hospitalisation once testing positive for Blacks, but a risk of testing positive outside hospital and testing negative similar to Whites. Our results also show increased testing rates among Black and Asian participants also supports studies undertaken in the UK using other data sources and methods, such as large scale primary care data[41].

[42][23]We found a test positivity rate similar to the general population but the case fatality rate seems to be higher than those of the general population for the same period. These differences are probably related to UK Biobank being composed of people of relatively older groups than the UK population.[42] We also found an increased mortality for those hospitalised with COVID-19 amongst Black, Asian and Chinese ethnic groups, although with high uncertainty due to low numbers. This is in line with previous studies, [9,18] but probably with a slightly lower due to those studies studying mortality in general population, and our lethality among the hospitalised.

Meaning of the study: possible explanations and implications for clinicians and policymakers

For peer review only

The underlying causes of the inequalities demonstrated by our study are likely to be multifactorial, complex, and varied.

Ethnic minority groups are disproportionately represented in lower socio-economic groups, and deprivation is an established driver of poorer health outcomes. In our data we could not disentangle deprivation effects from other related effects, like occupation, other household inhabitants occupation, or living conditions. Data from the US has suggested that ethnic hurminority groups are disproportionately represented in frontline roles which may increase exposure to the virus.[43] Similarly, in the UK, data from the Office for National Statistics has shown that 'key-workers' are more likely than average to be from an ethnic minority group.[44] There is also evidence suggesting that BAME groups in the UK are more likely to be employed in public-facing jobs where physical distancing is difficult (e.g., transport). [45,46] Finally, there is evidence that ethnic minorities are more likely to live in overcrowded households, which represents a risk factor for COVID-19 diagnosis and severity.

In addition, structural and cultural racism and discrimination are also likely to be important contributors to the adverse outcomes experienced by ethnic minority groups during the pandemic.[47] It is established that ethnic disparities exist in a wide range of other healthcare contexts, such as maternal deaths[48] and sectioning under the Mental Health Act.[49] This has been thought to be multifactorial. Practical aspects as transport availability and caring duties could have played a role. [50] Another possibly more important factor is racial discrimination in medical settings and other institutional barriers, as inadequate communication, and culturally insensitive care in the NHS. Studies have shown that implicit biases not only affect communication with patients, but also their experiences and outcomes of their clinical care.[51,52] This leads to lower trust in medical systems, and a higher susceptibility to targeted misinformation. [50,53,54] These inbuilt systemic biases towards ethnic minorities can be directly contributing to poorer outcomes after COVID-19 infection, due to delays in care, to the lower vaccination rate seen in the analyses, and to differential access to home testing and testing reporting.

Unanswered questions and future research

Further research using the datasets from this study, for example, could investigate availability of testing and vaccination by territorial region, which is a source of systemic bias. More research into the underlying causes of ethnic disparities in pandemic-related outcomes will be important, especially when designing practical, effective and appropriate public health policy responses to future pandemics.

For peer review only

Competing interests

Prof. Prieto-Alhambra's research group has received grant support from Amgen, Chesi-Taylor, Novartis, and UCB Biopharma. His department has received advisory or consultancy fees from Amgen, Astellas, AstraZeneca, Johnson, and Johnson, and UCB Biopharma and fees for speaker services from Amgen and UCB Biopharma. Janssen, on behalf of IMI-funded EHDEN and EMIF consortiums, and Synapse Management Partners have supported training programmes organised by DPA's department and open for external participants organized by his department outside submitted work.

Funding statement

The research was supported by the National Institute for Health Research (NIHR) Oxford Biomedical Research Centre (BRC). DPA is funded through a NIHR Senior Research Fellowship (Grant number SRF-2018-11-ST2-004). The views expressed in this publication are those of the author(s) and not necessarily those of the NHS, the National Institute for Health Research, or the Department of Health.

Data sharing statement

UK Biobank data are available under specific conditions directly from the UK Biobank platform: https://www.ukbiobank.ac.uk

Ethics approval

The North West Multi-center Research Ethics Committee (MREC) approved the collection and use of UK Biobank data (16/NW/0274). UKBiobank approved the application used to access the data (Application 46228) on 17/07/2019 and its access to COVID-19 data on 17/04/2020.

Statement of transparency

The lead author affirms that this manuscript is an honest, accurate, and transparent account of the study being reported; that no important aspects of the study have been omitted; and that any discrepancies from the study as planned (and, if relevant, registered) have been explained. Tomás Urdiales and Albert Prats-Uribe directly conducted the analyses and had access to the data.

Contributorship statement

DPA and CP conceived and designed the study. TU, APU and MC acquired and performed analyses of the data, and all authors were involved in interpretation of the findings. FD, TU and APU drafted the work, and all authors were involved in critical revisions of the subsequent drafts. All authors gave final approval of the version to be published, and agree to be accountable for all aspects of the work.

For peer review only

For peer review only

References

1 Dong E, Du H, Gardner L. An interactive web-based dashboard to track COVID-19 in real time. *Lancet Infect Dis* 2020;**20**:533–4. doi:10.1016/S1473-3099(20)30120-1

2 Sze S, Pan D, Nevill CR, *et al.* Ethnicity and clinical outcomes in COVID-19: A systematic review and meta-analysis. *EClinicalMedicine* 2020;**29–30**:100630. doi:10.1016/j.eclinm.2020.100630

3 Apea VJ, Wan YI, Dhairyawan R, *et al.* Ethnicity and outcomes in patients hospitalised with COVID-19 infection in East London: An observational cohort study. *BMJ Open* 2021;**11**:1–11. doi:10.1136/bmjopen-2020-042140

4 Sapey E, Gallier S, Mainey C, *et al.* Ethnicity and risk of death in patients hospitalised for COVID-19 infection in the UK: An observational cohort study in an urban catchment area. *BMJ Open Respir Res* 2020;**7**:1–11. doi:10.1136/bmjresp-2020-000644

5 Griffith GJ, Morris TT, Tudball MJ, *et al.* Collider bias undermines our understanding of COVID-19 disease risk and severity. *Nat Commun* 2020;**11**:1–12. doi:10.1038/s41467-020-19478-2

6 Sudlow C, Gallacher J, Allen N, *et al.* UK Biobank: An Open Access Resource for Identifying the Causes of a Wide Range of Complex Diseases of Middle and Old Age. *PLoS Med* 2015;**12**:1–10. doi:10.1371/journal.pmed.1001779

7 Armstrong J, Rudkin JK, Allen N, *et al.* Dynamic linkage of covid-19 test results between public health england's second generation surveillance system and uk biobank. *Microb Genom* 2020;**6**:1–9. doi:10.1099/mgen.0.000397

8 Atkins JL, Masoli JAH, Delgado J, *et al.* Preexisting Comorbidities Predicting COVID-19 and Mortality in the UK Biobank Community Cohort. *Journals of Gerontology - Series A Biological Sciences and Medical Sciences* 2020;**75**:2224–30. doi:10.1093/gerona/glaa183

9 Batty GD, Gaye B, Gale CR, *et al.* Explaining Ethnic Differentials in COVID-19 Mortality: A Cohort Study. *Am J Epidemiol* 2022;**191**:275–81. doi:10.1093/aje/kwab237

10 Patel AP, Paranjpe MD, Kathiresan NP, *et al.* Race, socioeconomic deprivation, and hospitalization for COVID-19 in English participants of a national biobank. *Int J Equity Health* 2020;**19**:1–4. doi:10.1186/s12939-020-01227-y

11 Raisi-Estabragh Z, McCracken C, Bethell MS, *et al.* Greater risk of severe COVID-19 in black, asian and minority ethnic populations is not explained by cardiometabolic, socioeconomic or behavioural factors, or by 25(OH)-vitamin D status: Study of 1326 cases from the UK biobank. *Journal of Public Health (United Kingdom)* 2020;**42**:451–60. doi:10.1093/pubmed/fdaa095

12 Razieh C, Zaccardi F, Gillies CL, *et al.* Ethnic minorities and COVID-19: examining whether excess risk is mediated through deprivation. *Eur J Public Health* 2021;**31**:630–4. doi:10.1093/eurpub/ckab041

13 Sattar N, Ho FK, Gill JM, *et al.* BMI and future risk for COVID-19 infection and death across sex, age and ethnicity: Preliminary findings from UK biobank. *Diabetes and Metabolic Syndrome: Clinical Research and Reviews* 2020;**14**:1149–51. doi:10.1016/j.dsx.2020.06.060

For peer review only

14 Woolford SJ, D'Angelo S, Curtis EM, *et al.* COVID-19 and associations with frailty and multimorbidity: a prospective analysis of UK Biobank participants. *Aging Clin Exp Res* 2020;**32**:1897–905. doi:10.1007/S40520-020-01653-6
15 Woodward M, Peters SAE, Harris K. Social deprivation as a risk factor for COVID-19 mortality among women and men in the UK Biobank: nature of risk and context suggests that social interventions are essential to mitigate the effects of future pandemics. *J Epidemiol Community Health (1978)* 2021;**75**. doi:10.1136/JECH-2020-215810
16 Chadeau-Hyam M, Bodinier B, Elliott J, *et al.* Risk factors for positive and negative COVID-19 tests: A cautious and in-depth analysis of UK biobank data. *Int J Epidemiol* 2020;**49**:1454–67. doi:10.1093/ije/dyaa134
17 Chudasama Y V., Zaccardi F, Gillies CL, *et al.* Patterns of multimorbidity and risk of severe SARS-CoV-2 infection: an observational study in the U.K. *BMC Infect Dis* 2021;**21**:1–12. doi:10.1186/s12879-021-06600-y
18 Elliott J, Bodinier B, Whitaker M, *et al.* COVID-19 mortality in the UK Biobank cohort: revisiting and evaluating risk factors. *Eur J Epidemiol* 2021;**36**:299–309. doi:10.1007/s10654-021-00722-y
19 Ho FK, Celis-Morales CA, Gray SR, *et al.* Modifiable and non-modifiable risk factors for COVID-19, and comparison to risk factors for influenza and pneumonia: Results from a UK Biobank prospective cohort study. *BMJ Open* 2020;**10**. doi:10.1136/bmjopen-2020-040402
20 Kolin DA, Kulm S, Christos PJ, *et al.* Clinical, regional, and genetic characteristics of Covid-19 patients from UK Biobank. *PLoS One* 2020;**15**:1–13. doi:10.1371/journal.pone.0241264
21 Lassale C, Gaye B, Hamer M, *et al.* Ethnic disparities in hospitalisation for COVID-19 in England: The role of socioeconomic factors, mental health, and inflammatory and pro-inflammatory factors in a community-based cohort study. *Brain Behav Immun* 2020;**88**:44–9. doi:10.1016/j.bbi.2020.05.074
22 McQueenie R, Foster HME, Jani BD, *et al.* Multimorbidity, polypharmacy, and COVID-19 infection within the UK Biobank cohort. *PLoS One* 2020;**15**:1–15. doi:10.1371/journal.pone.0238091
23 Niedzwiedz CL, O'Donnell CA, Jani BD, *et al.* Ethnic and socioeconomic differences in SARS-CoV-2 infection: Prospective cohort study using UK Biobank. *BMC Med* 2020;**18**:1–14. doi:10.1186/s12916-020-01640-8
24 UK Biobank. UK Biobank Mortality data: linkage to death registries. Published Online First: 2020.http://www.ukbiobank.ac.uk/ (accessed 2 Apr 2023).
25 Herbert A, Wijlaars L, Zylbersztejn A, *et al.* Data Resource Profile: Hospital Episode Statistics Admitted Patient Care (HES APC). *Int J Epidemiol* 2017;**46**:1093–1093i. doi:10.1093/IJE/DYX015
26 Meira-Machado LF, de Uña-Álvarez J, Cadarso-Suárez C, *et al.* Multi-state models for the analysis of time-to-event data. *Stat Methods Med Res* 2009;**18**:195–222. doi:10.1177/0962280208092301
27 Burn E, Tebé C, Fernandez-Bertolin S, *et al.* The natural history of symptomatic COVID-19 during the first wave in Catalonia. *Nat Commun* 2021;**12**. doi:10.1038/S41467-021-21100-Y
28 Flanagin A, Frey T, Christiansen SL. Updated Guidance on the Reporting of Race and Ethnicity in Medical and Science Journals. *JAMA* 2021;**326**:621–7. doi:10.1001/JAMA.2021.13304

For peer review only

29 English indices of deprivation 2019 - GOV.UK. https://www.gov.uk/government/statistics/english-indices-of-deprivation-2019 (accessed 2 Apr 2023).

30 Sundararajan V, Henderson T, Perry C, *et al.* New ICD-10 version of the Charlson comorbidity index predicted in-hospital mortality. *J Clin Epidemiol* 2004;**57**:1288–94. doi:10.1016/J.JCLINEPI.2004.03.012

31 Xie J, Prats-Uribe A, Feng Q, *et al.* Clinical and Genetic Risk Factors for Acute Incident Venous Thromboembolism in Ambulatory Patients With COVID-19. *JAMA Intern Med* 2022;**182**:1063–70. doi:10.1001/JAMAINTERNMED.2022.3858

32 Kaplan EL, Meier P. Nonparametric Estimation from Incomplete Observations. *J Am Stat Assoc* 1958;**53**:457. doi:10.2307/2281868

33 Wickham H, Averick M, Bryan J, *et al.* Wickham et al., (2019). Welcome to the tidyverse. Journal of Open Source Software, 4(43), 1686. https://doi.org/10.21105/joss.01686

34 de Wreede LC, Fiocco M, Putter H. mstate: An R Package for the Analysis of Competing Risks and Multi-State Models. *J Stat Softw* 2011;**38**:1–30. doi:10.18637/JSS.V038.I07

35 Therneau MT. Therneau T (2020). A Package for Survival Analysis in R. R package version 3.2-3. 2020;:1–172.Therneau T (2020). A Package for Survival Analysis in R. R package version 3.2-3

36 kableExtra: Construct Complex Table with 'kable' and Pipe Syntax version 1.3.4 from CRAN. https://rdrr.io/cran/kableExtra/ (accessed 2 Apr 2023).

37 Fry A, Littlejohns TJ, Sudlow C, *et al.* Comparison of Sociodemographic and Health-Related Characteristics of UK Biobank Participants With Those of the General Population. *Am J Epidemiol* 2017;**186**:1026–34. doi:10.1093/AJE/KWX246

38 Hastie CE, Mackay DF, Ho F, *et al.* Vitamin D concentrations and COVID-19 infection in UK Biobank. *Diabetes and Metabolic Syndrome: Clinical Research and Reviews* 2020;**14**:561–5. doi:10.1016/j.dsx.2020.04.050

39 Agyemang C, Richters A, Jolani S, *et al.* Ethnic minority status as social determinant for COVID-19 infection, hospitalisation, severity, ICU admission and deaths in the early phase of the pandemic: A meta-analysis. *BMJ Glob Health* 2021;**6**:1–14. doi:10.1136/bmjgh-2021-007433

40 Magesh S, John D, Li WT, *et al.* Disparities in COVID-19 Outcomes by Race, Ethnicity, and Socioeconomic Status: A Systematic-Review and Meta-analysis. *JAMA Netw Open* 2021;**4**:1–16. doi:10.1001/jamanetworkopen.2021.34147

41 Mathur R, Rentsch CT, Morton CE, *et al.* Ethnic differences in SARS-CoV-2 infection and COVID-19-related hospitalisation, intensive care unit admission, and death in 17 million adults in England: an observational cohort study using the OpenSAFELY platform. *The Lancet* 2021;**397**:1711–24. doi:10.1016/S0140-6736(21)00634-6

42 England Summary | Coronavirus (COVID-19) in the UK. UK Government. 2023.https://coronavirus.data.gov.uk/ (accessed 2 Apr 2023).

43 Goldman N, Pebley AR, Lee K, *et al.* Racial and ethnic differentials in COVID-19-related job exposures by occupational standing in the US. *PLoS One* 2021;**16**:1–17. doi:10.1371/journal.pone.0256085

44 https://commonslibrary.parliament.uk/coronavirus-which-key-workers-are-most-at-risk/.

For peer review only

45 Khunti K, Platt L, Routen A, *et al.* Covid-19 and ethnic minorities: an urgent agenda for overdue action The government's report falls seriously short on commitment. Published Online First: 2020. doi:10.1136/bmj.m2503

46 Magesh S, John D, Li WT, *et al.* Disparities in COVID-19 Outcomes by Race, Ethnicity, and Socioeconomic Status: A Systematic Review and Meta-analysis. *JAMA Netw Open* 2021;**4**:e2134147–e2134147. doi:10.1001/JAMANETWORKOPEN.2021.34147

47 Razai MS, Kankam HKN, Majeed A, *et al.* Mitigating ethnic disparities in covid-19 and beyond. *The BMJ* 2021;**372**:1–5. doi:10.1136/bmj.m4921

48 Knight M, Bunch K, Tuffnell D, *et al.* MBRRACE-UK Saving Lives, Improving Mothers' Care – Lessons learned to inform maternity care from the UK and Ireland Confidential Enquiries into Maternal Deaths and Morbidity 2017-19. Published Online First: 2011.https://www.npeu.ox.ac.uk/mbrrace-uk/reports

49 GOV.UK. Modernising the Mental Health Act - Final report of the Independent Review of the Mental Health Act 1983. 2018;:1–322.www.gov.uk/dhsc

50 Yong A, Germain S. Ethnic minority and migrant women's struggles in accessing healthcare during COVID-19: an intersectional analysis. *https://doi.org/101080/1479758520212012090* 2022;**26**:65–82. doi:10.1080/14797585.2021.2012090

51 Cooper LA, Roter DL, Carson KA, *et al.* The associations of clinicians' implicit attitudes about race with medical visit communication and patient ratings of interpersonal care. *Am J Public Health* 2012;**102**:979–87. doi:10.2105/AJPH.2011.300558

52 Van Ryn M, Burgess DJ, Dovidio JF, *et al.* The impact of racism on clinician cognition, behavior, and clinical decision making. *Du Bois Review* 2011;**8**:199–218. doi:10.1017/S1742058X11000191

53 Gaughan CH, Razieh C, Khunti K, *et al.* COVID-19 vaccination uptake amongst ethnic minority communities in England: a linked study exploring the drivers of differential vaccination rates. *J Public Health (Oxf)* 2023;**45**:e65–74. doi:10.1093/PUBMED/FDAB400

54 Paul E, Fancourt D, Razai M. Racial discrimination, low trust in the health system and COVID-19 vaccine uptake: a longitudinal observational study of 633 UK adults from ethnic minority groups. *J R Soc Med* 2022;**115**:439–47. doi:10.1177/01410768221095241

For peer review only

Figure legend

Figure 1. Overview of the multistate model used in this study with the four "COVID-19 states" and the possible transitions.

Figure 2. Flowchart outlining stepwise exclusions of participants

Figure 3. Forest plots of ethnicity-stratified hazard ratios, fully adjusted for age, sex and socioeconomic deprivation. The scale used for visualisation is linear but differs between each transition. The three dots reflect the x=1 axis.

For peer review only

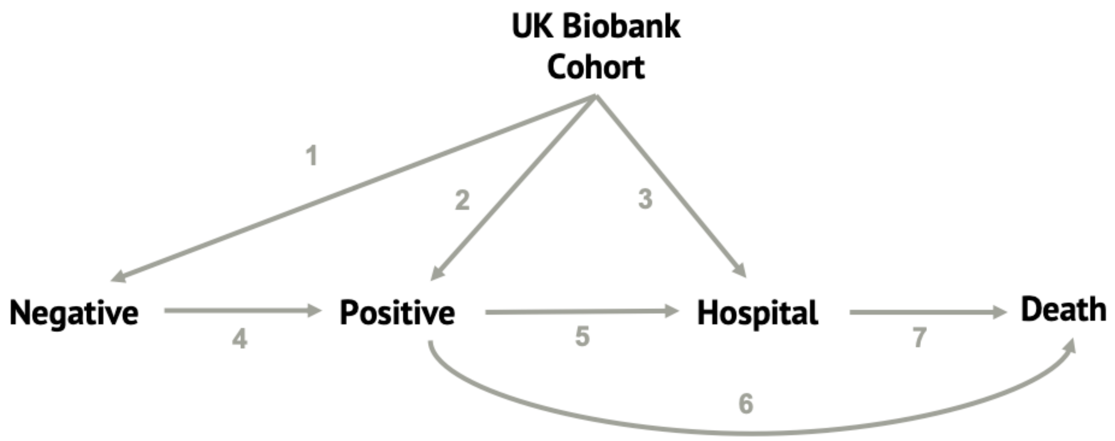

For peer review only

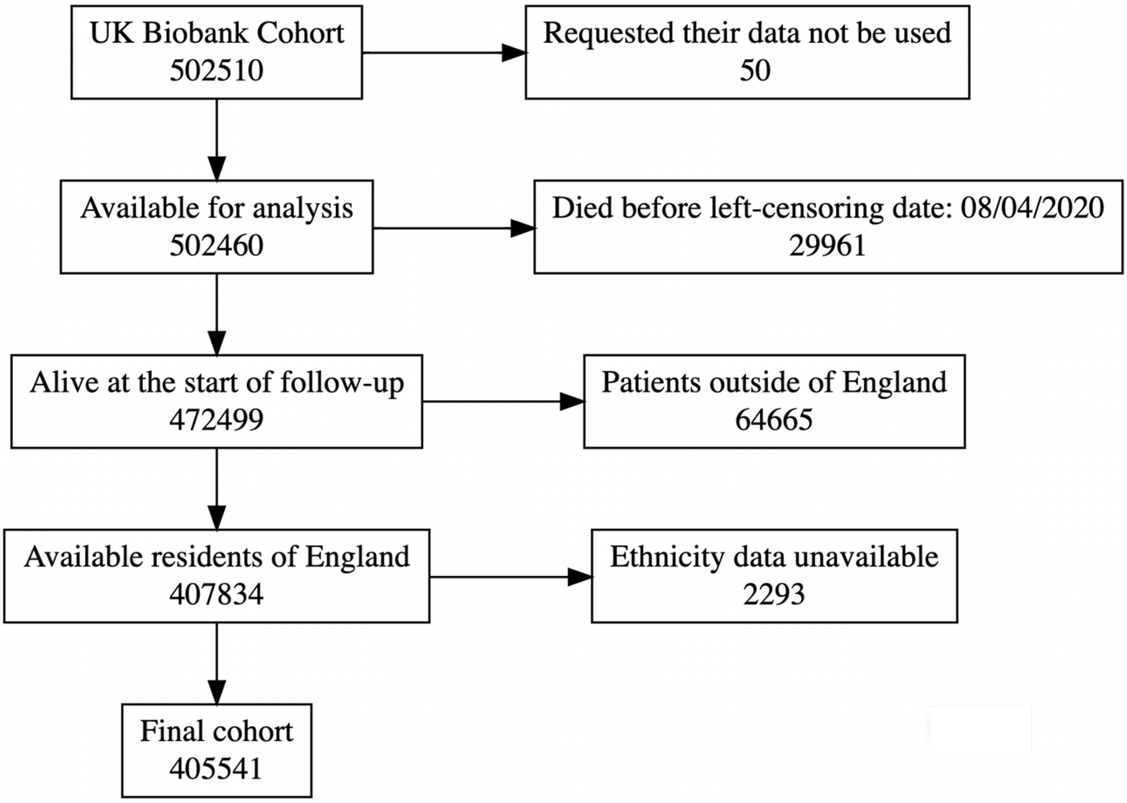
UK Biobank Cohort
502510
Requested their data not be used
50
Available for analysis
502460
Died before left-censoring date: 08/04/2020
29961
Alive at the start of follow-up
472499
Patients outside of England
64665
Available residents of England
407834
Ethnicity data unavailable
2293
Final cohort
405541

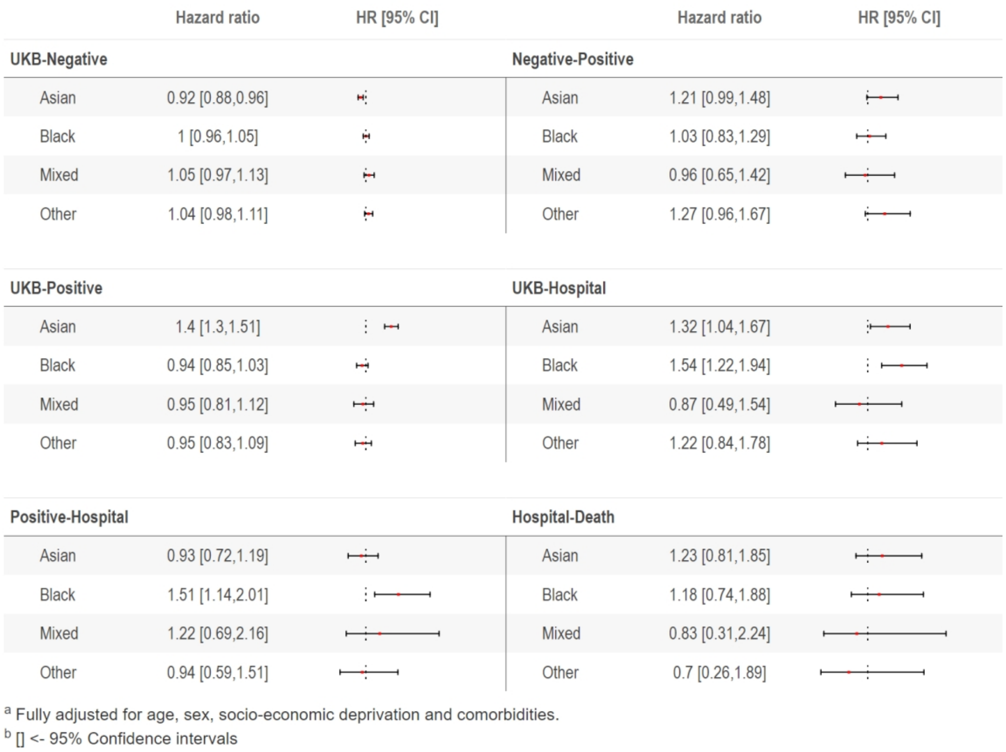

Forest plots of ethnicity-stratified hazard ratios, fully adjusted for age, sex, socioeconomic deprivation and comorbidities. The scale used for visualisation is linear but differs between each transition. The three dots reflect the x=1 axis.

854x653mm (47 x 47 DPI)

Supplementary Figure 1. Radar charts for socio-economic deprivation quintile population distribution, stratified by ethnicity. The distribution of each ethnic group is shown by the coloured pentagons, in comparison to the whole cohort distribution in dark grey beneath.

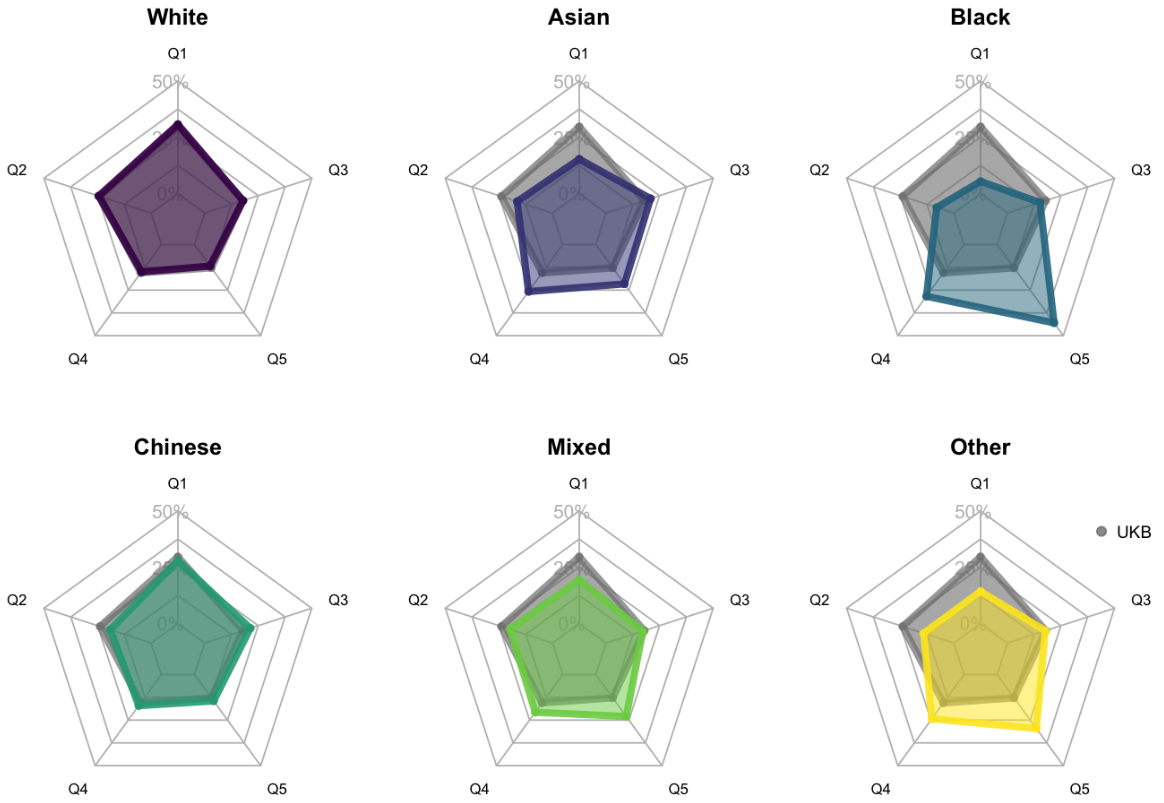

w only

Supplementary Table 1. COVID outcome event numbers. Stratified by ethnicity, socioeconomic deprivation quintile, sex and age. All percentages (in brackets) are calculated as the fraction of individuals who underwent a particular transition from the total who were in the states preceding it (and thus susceptible to transitioning). Age is divided into those younger and older than the median age 70.8.

	Total	UKB-Negative	Negative-Positive	UKB-Positive	Positive-Hospital	Positive-Death	UKB-Hospital	Hospital-Death
All (%)	405541	111614 (27.5)	3285 (2.9)	18172 (4.5)	1490 (6.9)	129 (0.6)	2322 (0.6)	662 (17.4)
**Ethnicity**								
Asian (%)	8791	2132 (24.3)	100 (4.7)	714 (8.1)	68 (8.4)	4 (0.5)	75 (0.9)	25 (17.5)
Black (%)	7275	1883 (25.9)	84 (4.5)	450 (6.2)	52 (9.7)	3 (0.6)	82 (1.1)	19 (14.2)
Chinese (%)	1324	238 (18)	4 (1.7)	31 (2.3)	3 (8.6)	0 (0)	7 (0.5)	2 (20)
Mixed (%)	2537	688 (27.1)	25 (3.6)	146 (5.8)	12 (7)	0 (0)	12 (0.5)	4 (16.7)
Other (%)	3964	1068 (26.9)	53 (5)	226 (5.7)	18 (6.5)	2 (0.7)	28 (0.7)	4 (8.7)
White (%)	381650	105605 (27.7)	3019 (2.9)	16605 (4.4)	1337 (6.8)	120 (0.6)	2118 (0.6)	608 (17.6)
**Socio-economic deprivation**								
Q1 (%)	120443	34121 (28.3)	790 (2.3)	4112 (3.4)	290 (5.9)	34 (0.7)	478 (0.4)	127 (16.5)
Q2 (%)	97002	26912 (27.7)	741 (2.8)	4006 (4.1)	258 (5.4)	20 (0.4)	431 (0.4)	95 (13.8)
Q3 (%)	72708	19607 (27)	585 (3)	3454 (4.8)	286 (7.1)	18 (0.4)	391 (0.5)	132 (19.5)
Q4 (%)	63404	17235 (27.2)	615 (3.6)	3363 (5.3)	294 (7.4)	30 (0.8)	461 (0.7)	138 (18.3)
Q5 (%)	51984	13739 (26.4)	554 (4)	3237 (6.2)	362 (9.5)	27 (0.7)	561 (1.1)	170 (18.4)
**Sex**								

	Total	UKB-Negative	Negative-Positive	UKB-Positive	Positive-Hospital	Positive-Death	UKB-Hospital	Hospital-Death
Male (%)	181413	51172 (28.2)	1512 (3)	8365 (4.6)	829 (8.4)	72 (0.7)	1281 (0.7)	429 (20.3)
Female (%)	224128	60442 (27)	1773 (2.9)	9807 (4.4)	661 (5.7)	57 (0.5)	1041 (0.5)	233 (13.7)
**Age**								
Younger than 70 (%)	201131	51280 (12.6)	1970 (1.8)	12598 (3.1)	729 (3.4)	16 (0.1)	725 (0.2)	129 (3.4)
Older than 70 (%)	204410	60334 (14.9)	1315 (1.2)	5574 (1.4)	761 (3.5)	113 (0.5)	1597 (0.4)	533 (14)

For peer review only

Supplementary Table 2. Hazard ratio coefficients stratified by ethnicity, at different levels of confounding. Fully adjusted for age, sex, socioeconomic deprivation and comorbidities.

	UKB-Negative	Negative-Positive	UKB-Positive	Positive-Hospital	UKB-Hospital	Hospital-Death
**Asian vs White**						
Unadjusted	0.9 [0.86,0.93]	1.54 [1.26,1.88]	1.91 [1.77,2.06]	1.05 [0.82,1.34]	1.56 [1.24,1.97]	0.93 [0.62,1.39]
Age-sex adjusted	0.94 [0.9,0.98]	1.3 [1.07,1.59]	1.55 [1.44,1.67]	1.26 [0.99,1.61]	1.89 [1.5,2.39]	1.34 [0.9,2.01]
Fully adjusted	0.92 [0.88,0.96]	1.21 [0.99,1.48]	1.4 [1.3,1.51]	0.93 [0.72,1.19]	1.32 [1.04,1.67]	1.23 [0.81,1.85]
**Black vs White**						
Unadjusted	0.95 [0.91,1]	1.48 [1.2,1.84]	1.44 [1.31,1.58]	1.44 [1.09,1.9]	2 [1.6,2.51]	0.82 [0.52,1.3]
Age-sex adjusted	1.02 [0.98,1.07]	1.17 [0.94,1.45]	1.11 [1.01,1.22]	1.96 [1.48,2.59]	2.79 [2.22,3.49]	1.25 [0.79,1.98]
Fully adjusted	1 [0.96,1.05]	1.03 [0.83,1.29]	0.94 [0.85,1.03]	1.51 [1.14,2.01]	1.54 [1.22,1.94]	1.18 [0.74,1.88]
**Chinese vs White**						
Unadjusted	0.6 [0.53,0.69]	0.61 [0.23,1.62]	0.5 [0.35,0.71]	1.28 [0.41,3.99]	0.91 [0.43,1.91]	0.77 [0.19,3.1]
Age-sex adjusted	0.64 [0.57,0.73]	0.51 [0.19,1.36]	0.41 [0.29,0.58]	1.9 [0.61,5.92]	1.24 [0.59,2.61]	1.1 [0.27,4.4]
Fully adjusted	0.67 [0.59,0.76]	0.5 [0.19,1.33]	0.41 [0.29,0.58]	1.78 [0.57,5.56]	1.32 [0.63,2.77]	1.21 [0.3,4.87]
**Mixed vs White**						
Unadjusted	0.99 [0.92,1.06]	1.29 [0.87,1.91]	1.33 [1.13,1.57]	1 [0.57,1.76]	0.87 [0.49,1.53]	0.85 [0.32,2.27]

	UKB-Negative	Negative-Positive	UKB-Positive	Positive-Hospital	UKB-Hospital	Hospital-Death
Age-sex adjusted	1.07 [0.99,1.15]	1 [0.68,1.49]	1.02 [0.87,1.2]	1.41 [0.8,2.5]	1.24 [0.7,2.18]	1.03 [0.39,2.77]
Fully adjusted	1.05 [0.97,1.13]	0.96 [0.65,1.42]	0.95 [0.81,1.12]	1.22 [0.69,2.16]	0.87 [0.49,1.54]	0.83 [0.31,2.24]
**Other vs White**						
Unadjusted	0.98 [0.93,1.04]	1.76 [1.34,2.31]	1.32 [1.16,1.5]	0.84 [0.52,1.33]	1.3 [0.89,1.88]	0.51 [0.19,1.36]
Age-sex adjusted	1.04 [0.98,1.11]	1.41 [1.07,1.85]	1.06 [0.93,1.21]	1.08 [0.68,1.72]	1.72 [1.18,2.49]	0.76 [0.28,2.02]
Fully adjusted	1.04 [0.98,1.11]	1.27 [0.96,1.67]	0.95 [0.83,1.09]	0.94 [0.59,1.51]	1.22 [0.84,1.78]	0.7 [0.26,1.89]

[a] Fully adjusted for age, sex, socio-economic deprivation and comorbidities.

[b] [] <- 95% Confidence intervals

For peer review only

For peer review only

The RECORD statement – checklist of items, extended from the STROBE statement, that should be reported in observational studies using routinely collected health data.

	Item No.	STROBE items	Location in manuscript where items are reported	RECORD items	Location in manuscript where items are reported
**Title and abstract**					
	1	(a) Indicate the study's design with a commonly used term in the title or the abstract (b) Provide in the abstract an informative and balanced summary of what was done and what was found	Abstract (page 1)	RECORD 1.1: The type of data used should be specified in the title or abstract. When possible, the name of the databases used should be included.<br><br>RECORD 1.2: If applicable, the geographic region and timeframe within which the study took place should be reported in the title or abstract.<br><br>RECORD 1.3: If linkage between databases was conducted for the study, this should be clearly stated in the title or abstract.	Abstract (page 1)<br><br>Abstract (page 1)
**Introduction**					
Background rationale	2	Explain the scientific background and rationale for the investigation being reported	Introduction (page 3)		
Objectives	3	State specific objectives, including any prespecified hypotheses	Introduction (page 3)		
**Methods**					
Study Design	4	Present key elements of study design early in the paper	Methods (page 3-5)		
Setting	5	Describe the setting, locations, and relevant dates, including periods of recruitment, exposure, follow-up, and data collection	Methods (page 3-5)		

For peer review only

Participants	6	*(a) Cohort study* - Give the eligibility criteria, and the sources and methods of selection of participants. Describe methods of follow-up<br>*Case-control study* - Give the eligibility criteria, and the sources and methods of case ascertainment and control selection. Give the rationale for the choice of cases and controls<br>*Cross-sectional study* - Give the eligibility criteria, and the sources and methods of selection of participants<br><br>*(b) Cohort study* - For matched studies, give matching criteria and number of exposed and unexposed<br>*Case-control study* - For matched studies, give matching criteria and the number of controls per case	Methods (page 3-5)	RECORD 6.1: The methods of study population selection (such as codes or algorithms used to identify subjects) should be listed in detail. If this is not possible, an explanation should be provided.<br><br>RECORD 6.2: Any validation studies of the codes or algorithms used to select the population should be referenced. If validation was conducted for this study and not published elsewhere, detailed methods and results should be provided.<br><br>RECORD 6.3: If the study involved linkage of databases, consider use of a flow diagram or other graphical display to demonstrate the data linkage process, including the number of individuals with linked data at each stage.	Methods (page 3-5)
Variables	7	Clearly define all outcomes, exposures, predictors, potential confounders, and effect modifiers. Give diagnostic criteria, if applicable.	Methods (page 3-5)	RECORD 7.1: A complete list of codes and algorithms used to classify exposures, outcomes, confounders, and effect modifiers should be provided. If these cannot be reported, an explanation should be provided.	Methods (page 3-5)
Data sources/ measurement	8	For each variable of interest, give sources of data and details of methods of assessment (measurement).<br>Describe comparability of assessment methods if there is more than one group	Methods (page 3-5)		

Bias	9	Describe any efforts to address potential sources of bias	Methods (page 3-5)		
Study size	10	Explain how the study size was arrived at	Methods (page 3-5)		
Quantitative variables	11	Explain how quantitative variables were handled in the analyses. If applicable, describe which groupings were chosen, and why	Methods (page 3-5)		
Statistical methods	12	(a) Describe all statistical methods, including those used to control for confounding<br>(b) Describe any methods used to examine subgroups and interactions<br>(c) Explain how missing data were addressed<br>(d) *Cohort study* - If applicable, explain how loss to follow-up was addressed<br>*Case-control study* - If applicable, explain how matching of cases and controls was addressed<br>*Cross-sectional study* - If applicable, describe analytical methods taking account of sampling strategy<br>(e) Describe any sensitivity analyses	Methods (page 3-5)		
Data access and cleaning methods		..		RECORD 12.1: Authors should describe the extent to which the investigators had access to the database population used to create the study population.	Transparency (page 12) and Methods (page 3-5)

For peer review only

				RECORD 12.2: Authors should provide information on the data cleaning methods used in the study.	
Linkage		..		RECORD 12.3: State whether the study included person-level, institutional-level, or other data linkage across two or more databases. The methods of linkage and methods of linkage quality evaluation should be provided.	Methods (page 3-5)
**Results**					
Participants	13	(a) Report the numbers of individuals at each stage of the study (*e.g.*, numbers potentially eligible, examined for eligibility, confirmed eligible, included in the study, completing follow-up, and analysed)<br>(b) Give reasons for non-participation at each stage.<br>(c) Consider use of a flow diagram	Results (page 6)	RECORD 13.1: Describe in detail the selection of the persons included in the study (*i.e.,* study population selection) including filtering based on data quality, data availability and linkage. The selection of included persons can be described in the text and/or by means of the study flow diagram.	Methods (page 3-5)
Descriptive data	14	(a) Give characteristics of study participants (*e.g.*, demographic, clinical, social) and information on exposures and potential confounders<br>(b) Indicate the number of participants with missing data for each variable of interest<br>(c) *Cohort study* - summarise follow-up time (*e.g.*, average and total amount)	Results (page 6)		
Outcome data	15	*Cohort study* - Report numbers of outcome events or summary measures over time<br>*Case-control study* - Report numbers in each exposure	Results (page 7)		

For peer review only

		category, or summary measures of exposure<br>*Cross-sectional study* - Report numbers of outcome events or summary measures			
Main results	16	(a) Give unadjusted estimates and, if applicable, confounder-adjusted estimates and their precision (e.g., 95% confidence interval). Make clear which confounders were adjusted for and why they were included<br>(b) Report category boundaries when continuous variables were categorized<br>(c) If relevant, consider translating estimates of relative risk into absolute risk for a meaningful time period	Results (page 6-9)		
Other analyses	17	Report other analyses done—e.g., analyses of subgroups and interactions, and sensitivity analyses	Results (page 6-9)		
**Discussion**					
Key results	18	Summarise key results with reference to study objectives	Discussion (page 10-11)		
Limitations	19	Discuss limitations of the study, taking into account sources of potential bias or imprecision. Discuss both direction and magnitude of any potential bias	Discussion (page 10-11)	RECORD 19.1: Discuss the implications of using data that were not created or collected to answer the specific research question(s). Include discussion of misclassification bias, unmeasured confounding, missing data, and changing eligibility over time, as they pertain to the study being reported.	Discussion (page 10-11)
Interpretation	20	Give a cautious overall interpretation of results considering objectives,	Discussion (page 10-11)		

For peer review only

		limitations, multiplicity of analyses, results from similar studies, and other relevant evidence			
Generalisability	21	Discuss the generalisability (external validity) of the study results	Discussion (page 10-11)		
**Other Information**					
Funding	22	Give the source of funding and the role of the funders for the present study and, if applicable, for the original study on which the present article is based	Other information, at end of manuscript (page 12)		
Accessibility of protocol, raw data, and programming code		..		RECORD 22.1: Authors should provide information on how to access any supplemental information such as the study protocol, raw data, or programming code.	Methods (page 3-5)

*Reference: Benchimol EI, Smeeth L, Guttmann A, Harron K, Moher D, Petersen I, Sørensen HT, von Elm E, Langan SM, the RECORD Working Committee. The REporting of studies Conducted using Observational Routinely-collected health Data (RECORD) Statement. *PLoS Medicine* 2015; in press.

*Checklist is protected under Creative Commons Attribution (CC BY) license.

For peer review only

BMJ Open

The association between ethnic background and COVID-19 morbidity, mortality and vaccination in England: a multi-state cohort analysis using the UK Biobank

Journal:	*BMJ Open*
Manuscript ID	bmjopen-2023-074367.R1
Article Type:	Original research
Date Submitted by the Author:	19-Jul-2023
Complete List of Authors:	Urdiales, Tomás; Universitat Politècnica de Catalunya, Physics Department; Royal Institute of Technology, Department of Energy Technolgy<br>Dernie, Francesco; University of Oxford,<br>Català, Martí; University of Oxford, Pharmaco- and Device Epidemiology, Centre for Statistics in Medicine, Nuffield Department of Orthopaedics, Rheumatology and Musculoskeletal Sciences<br>Prats-Uribe, Albert; University of Oxford Centre for Statistics in Medicine, Nuffield Department of Orthopaedics Rheumatology and Musculoskeletal Science<br>Prats, Clara; Universitat Politècnica de Catalunya, Physics Department<br>Prieto-Alhambra, Daniel; University of Oxford Centre for Statistics in Medicine, NDORMS
**Primary Subject Heading**:	Epidemiology
Secondary Subject Heading:	Public health, Epidemiology, Infectious diseases, Respiratory medicine
Keywords:	COVID-19, Public health < INFECTIOUS DISEASES, Epidemiology < INFECTIOUS DISEASES

SCHOLARONE™
Manuscripts

I, the Submitting Author has the right to grant and does grant on behalf of all authors of the Work (as defined in the below author licence), an exclusive licence and/or a non-exclusive licence for contributions from authors who are: i) UK Crown employees; ii) where BMJ has agreed a CC-BY licence shall apply, and/or iii) in accordance with the terms applicable for US Federal Government officers or employees acting as part of their official duties; on a worldwide, perpetual, irrevocable, royalty-free basis to BMJ Publishing Group Ltd ("BMJ") its licensees and where the relevant Journal is co-owned by BMJ to the co-owners of the Journal, to publish the Work in this journal and any other BMJ products and to exploit all rights, as set out in our licence.

The Submitting Author accepts and understands that any supply made under these terms is made by BMJ to the Submitting Author unless you are acting as an employee on behalf of your employer or a postgraduate student of an affiliated institution which is paying any applicable article publishing charge ("APC") for Open Access articles. Where the Submitting Author wishes to make the Work available on an Open Access basis (and intends to pay the relevant APC), the terms of reuse of such Open Access shall be governed by a Creative Commons licence – details of these licences and which Creative Commons licence will apply to this Work are set out in our licence referred to above.

Other than as permitted in any relevant BMJ Author's Self Archiving Policies, I confirm this Work has not been accepted for publication elsewhere, is not being considered for publication elsewhere and does not duplicate material already published. I confirm all authors consent to publication of this Work and authorise the granting of this licence.

For peer review only

The association between ethnic background and COVID-19 morbidity, mortality and vaccination in England: a multi-state cohort analysis using the UK Biobank

AUTHORS: Tomás Urdiales[1,2], Francesco Dernie[3], Martí Català[4], Albert Prats-Uribe[4]*, Clara Prats[1], and Daniel Prieto-Alhambra[4,5]**

*Correspondence to: Albert Prats-Uribe (albert.prats-uribe@ndorms.ox.ac.uk)
** Senior author

1. Physics Department, Universitat Politècnica de Catalunya, Barcelona, Spain.

2. Department of Energy Technology, Royal Institute of Technology (KTH), Stockholm, Sweden.

3. University of Oxford Medical School, Medical Sciences Divisional Office, University of Oxford, Oxford, OX3 9DU, UK.

4. Pharmaco- and Device Epidemiology, Centre for Statistics in Medicine, Nuffield Department of Orthopaedics, Rheumatology and Musculoskeletal Sciences, University of Oxford, Oxford, UK.

5. Department of Medical Informatics, Erasmus Medical Centre University, Rotterdam, Netherlands

For peer review only

Abstract

Objectives: Despite growing evidence suggesting increased COVID-19 mortality among people from ethnic minorities, little is known about milder forms of SARS-CoV-2 infection. We sought to explore the association between ethnic background and the probability of testing, testing positive, hospitalisation, COVID-19 mortality, and vaccination uptake.

Design: A multi-state cohort analysis. Participants were followed between 8th April 2020 to 30th September 2021.

Setting: The UK Biobank, which stores medical data on around half a million people who were recruited between 2006-2010.

Participants: 405,541 subjects were eligible for analysis, limited to UK Biobank participants living in England. 23,891 (6%) of participants were non-white.

Primary and secondary outcome measures: The associations between ethnic background and testing, testing positive, hospitalisation and COVID-19 mortality were studied using multi-state survival analyses. The association with single and double-dose vaccination was also modelled. Multi-state models adjusted for age, sex, and socio-economic deprivation were fitted to estimate adjusted Hazard Ratios (aHR) for each of the multi-state transitions

Results: 18,172 (4.5%) individuals tested positive, 3,285 (0.8%) tested negative and then positive, 1,490 (6.9% of those tested positive) were hospitalised, and 129 (0.6%) tested positive at the moment of hospital admission (i.e., direct hospitalisation). Finally, 662 (17.4%) died after admission. Compared to White participants, Asian participants had an increased risk of negative to positive transition (aHR 1.24 [1.02-1.52]), testing positive (1.44 [1.33-1.55]) and direct hospitalisation (1.61 [1.28-2.03]). Black participants had an increased risk of hospitalisation following a positive test (1.71 [1.29-2.27]) and direct hospitalisation (1.90 [1.51-2.39]). Although not the case for Asians (aHR 1.00 [0.98-1.02]), Black participants had a reduced vaccination probability (0.63 [0.62-0.65]). In contrast, Chinese participants had a reduced risk of testing negative (aHR 0.64 [0.57-0.73]), of testing positive (0.40 [0.28-0.57]), and of vaccination (0.78 [0.74-0.83]).

Conclusions: We identified inequities in testing, vaccination and COVID-19 outcomes according to ethnicity in England. Compared to Whites, Asian participants had increased risks of infection and admission, and Black participants had almost double hospitalisation risk, and a 40% lower vaccine uptake.

For peer review only - http://bmjopen.bmj.com/site/about/guidelines.xhtml

Strengths and limitations of this study

- This study uses a large cohort of adults, allowing for high powered assessment of associations between baseline characteristics and COVID outcomes
- Key confounders, both individual-level and ecological, are adjusted for in our statistical analysis
- Our multistate analyses are able to take into account differential confounding structure for each transition.
- The UK Biobank cohort is not optimally representative, limiting extrapolation of findings to the wider population

For peer review only

Introduction

Since the first cases of human infection with SARS-CoV-2 were reported in Wuhan, China in late 2019, as of the 15th of March 2022 the COVID-19 pandemic has seen more than 450 million confirmed cases and 6 million confirmed deaths worldwide, causing unprecedented economic and social disruption.[1] Despite its global reach, it has become increasingly clear that the risks of initial infection, death and long-term consequences from COVID-19 are not distributed equally in society, including between different ethnic groups.[2]

In the early phase of the pandemic, initial studies sought to characterise factors associated with COVID infection in patients admitted to hospital,[3,4] as only these patients received formal COVID tests at the time. However, by focussing on this limited patient group, these studies had intrinsic biases which limited their generalisability to the wider population.[5]

To address these concerns, studies have sought to study COVID-19 infection risk in larger population cohorts, such as the established UK Biobank project.[6] Early in the pandemic, COVID-19 test results for UK Biobank participants were linked to their biobank data, allowing participants' COVID status to be followed.[7] These studies have identified several factors, including age, sex, comorbidities, ethnicity and socioeconomic status as significant determinants of COVID-19 infection, hospitalisation, and mortality.[8–23] Specifically, studies have suggested that Black and Asian people are at increased risk of testing positive, being hospitalised and dying from COVID-19, compared to White people. [9–12,16,18–21,23]

However, these studies were also prone to limitations. Firstly, many gathered information from the UK Biobank only during the first wave of COVID-19 (February-May 2020), meaning there were relatively few positive cases to compare to the rest of the cohort, and wider population testing (so-called 'pillar 2' testing) only became available in the UK in early April 2020. Secondly, many only dealt with individual outcome measures – either testing positive, being hospitalised, or death – meaning that the rest of the individual's journey through COVID-19 was not comprehensively assessed. This lack of detail may mask some differences between groups, thus limiting the implementation of findings concerning the role of ethnicity into public health policy in both the current and future pandemics. Furthermore, the lack of a multistate analysis could increase the chances of bias and erroneous conclusions, especially when focusing on ethnicity where inequities can lead to differential risks of transitions between testing, infection, hospitalisation, and death.

This paper seeks to address several of these issues to delineate the influence of ethnicity on a variety of COVID-19 outcomes. We use UK Biobank data from the start of pillar 2 testing (April 2020) until September 2021, allowing an up-to-date comparison of a higher number of positive cases, across multiple waves of the pandemic. We analyse this data using a multistate model to be able to study participants' transitions between different 'COVID states'. given that the outcome of interest was just the first transition into any of the stage of a journey through COVID-19.

For peer review only - http://bmjopen.bmj.com/site/about/guidelines.xhtml

Methods

Study design, setting and data sources

We conducted a cohort study using the UK Biobank cohort[5], a large, non-commercial, long-term biobank project in the United Kingdom which stores medical data on around half a million people who were recruited between 2006-2010. All participants were aged between 40-69 years at the time of their registration with the project and will remain in follow-up for a minimum of 30 years. All participants provided demographic and lifestyle information, as well as blood, urine, and saliva samples. COVID-19 testing data was provided by the UK Biobank through dynamic linkage with Public Health England's Second Generation Surveillance System.[7] This data is only available for England and has 108 laboratories reporting positive test, and 101 reporting negative tests too. Data on deaths was taken from the Office of National Statistics, which the UK Biobank updates regularly for their participants., and it is fully linked for England. [24]

We followed participants in this programme from the 8th April 2020 to 30th September 2021, which is representative of the start of wider population testing (pillar 2) for COVID-19 in the UK to the latest available data entry point common to all different data sources (testing, hospitalisation, mortality records) at the time of analysis and writing. We limited our analysis to participants in England, given that England has a different multiple economic deprivation index to the other constituent countries of the UK, and testing data was only available for England.

Data from the UK Biobank was combined with National Health Service (NHS) Hospital Episode Statistics (HES) Admitted Patient Care (APC) data,[25] a programme which stores data on all hospital admissions, outpatient appointments and attendances, to build a comprehensive database that gathered information on COVID-19 testing, hospitalisation and mortality, as well as key variables including age, sex, ethnicity, and socioeconomic status for the entire cohort.

Multistate framework

A multi-state model [26] served as the framework for analysis, which has been used previously to describe risks of COVID-19 diagnosis, hospitalisation and death elsewhere.[27] These models allow the characterisation and analysis of individual transitions between different 'COVID states'. In this study, all participants began as part of the UK Biobank Cohort (UKB), who had never been tested for COVID-19. They could then progress to one of three separate states: testing negative, testing positive, or direct hospitalisation (where testing positive for COVID-19 occurred on the same day as hospitalisation). Addition of a fifth health state of 'death' allowed for seven separate possible transitions: UKB to negative, UKB to positive, UKB to hospital, negative to positive, positive to hospital, positive to death (where

For peer review only

hospitalisation occurred on the same day as death, or the patient was never hospitalised), and hospital to death (Figure 1). Only the first negative test and subsequent positive test, or the first positive test, were counted, and each transition is unidirectional, so once an individual had a positive test, any subsequent negative tests are discounted.

Participants and study size

We initially included all participants of the UK Biobank programme. We excluded participants if they live outside of England, had died prior to the start of follow-up (8th April 2020), had requested that their data not be used for research, or had either missing data on ethnic background or had answered 'Do not know' or 'Prefer not to say' to questions asking about ethnic background.

Variables

The main 'exposure' of interest was ethnicity. Ethnicity was categorised into six groups, in line with the ethnic groupings specified by the UK Biobank at the time of recruitment to their programme. These are: Asian, Black, Chinese, Mixed, White and 'other'. Further detail on the ethnicities comprising each group can be found in the UKBiobank showcase (https://biobank.ndph.ox.ac.uk/showcase/field.cgi?id=21000).Language used in the reporting and discussion of ethnicity in the text is in line with published recommendations.[28] The main covariates for multivariable adjustment were sex, age and socioeconomic deprivation (Index of Multiple Deprivation quintiles). and comorbidities The Index of Multiple Deprivation combines information from seven unique domains of deprivation, to provide an overall relative measure of deprivation for 32,844 neighbourhoods in England (Lower-layer Super Output Areas). [29] Comorbidities were assessed using both hospitalisation data (HES) and Primary Care at the time of start of follow-up (8th April 2020). We used ICD-10 for HES,[30] and SNOMED and READ codes for primary care used previously,[31] using the categories of the Charlson comorbidity index.

Outcomes

The outcomes of interest were results of COVID tests, hospitalisation with COVID-19 and COVID-19-related death, gathered using the data sources discussed above. We deemed as COVID-19 related death or hospitalisation with COVID those which occurred within 28 days of a positive SARS-CoV-2 test. COVID-19 was not required to be the primary diagnosis or reason for hospitalisation. Data on whether tests were positive or not was obtained from Public Health England as described elsewhere.[7]

Test Count: Another outcome of interest was the test count, defined as the number of tests taken for each patient since the start of the follow-up period until the end of it.

Receiving a COVID-19 vaccine was defined as having a code READ code "Y29e7" or SNOMED CT "1324681000000101" for the first dose and "Y29e8" or "1324691000000104" for the second dose on the Primary Care data.

For peer review only - http://bmjopen.bmj.com/site/about/guidelines.xhtml

Statistical methods

Baseline characteristics are presented as n (%) or median (IQR) overall and for each ethnicity category. Incidence of outcomes are shown as % of people who transition from one state to another during the whole study period. A Cox proportional hazards model was used to determine hazard ratios for each group undergoing each transition in the multi-state model, adjusted for the confounders outlined above. A 'clock forward' approach, modelling all transitions in the same timescale (time since entering the study), for time-to-events was used. The proportional hazards assumption was checked using the Kaplan-Meier estimator.[32]

Quasi-Poisson (to account for overdispersion) regressions were used to analyse the number of tests undertaken by each participant. It was further stratified by ethnic background.

For the analyses of vaccines, a Cox proportional hazards models was again used to determine hazard ratios for each variable and transition: from unvaccinated to first dose, and from first dose to second dose.

All statistical analyses were carried out using R: the *tidyverse* collection of packages for data curation,[33] the *mstate* and *survival* packages for multistate modelling and statistical regressions,[34,35] and *kable* and *kableExtra* for graphical representation. [36]

Hazard ratios for the Positive to Death transition are not shown, as most groups had no individuals undergoing this transition, causing confidence intervals which were too wide to provide real information about relative risk.

Patient and public involvement

No funding was available for patient or public involvement in this project. No patients were involved in setting the research question or the outcome measures. Patients were not invited to comment on the study design, not consulted to develop patient relevant outcomes or interpret the results, and not invited to contribute to the writing or editing of this document for readability or accuracy.

Results

Baseline characteristics

After initial inclusion of all participants of the UK Biobank programme (n=502,510), a total of 405,541 (80.7%) participants were included in the final analysis (Figure 2). The baseline characteristics of the final population are summarised in Table 1. Our cohort was predominantly White with 381,650 (94.1%), and the majority were female (224,128 - 55.3%). The median age was 70.5 years old, but the median age of White participants was around five years older than participants of other ethnic groups. Participants living in areas in the upper quintiles of socio-economic status were more frequent in the cohort overall (29.7% in Q1 vs

For peer review only - http://bmjopen.bmj.com/site/about/guidelines.xhtml

12.8% in Q5), and there was clear disparity in the distribution of participants between different quintiles based on ethnicity (Supplementary Figure 1).

Table 1. Cohort population baseline characteristics. Data is stratified by ethnicity and displays sex, age, socio-economic status and comorbidities. Age is presented by group median age alongside interquartile range. All percentages (in brackets) are calculated with respect to group size *n*.

	All	Asian	Black	Chinese	Mixed	Other	White
**n**	405541	8791	7275	1324	2537	3964	381650
**Sex=Male (%)**	181413 (44.7)	4642 (52.8)	3017 (41.5)	468 (35.3)	925 (36.5)	1685 (42.5)	170676 (44.7)
**Age (median [IQR])**	70.5 [62.8,75.8]	65.2 [58.7,72.5]	63 [57.9,70]	65.1 [58.9,70.9]	62.8 [57.6,70.4]	64.6 [58.3,71.3]	70.8 [63.2,76.1]
**Socio-economic deprivation**							
**Q1 (%)**	120443 (29.7)	1345 (15.3)	390 (5.4)	370 (27.9)	492 (19.4)	567 (14.3)	117279 (30.7)
**Q2 (%)**	97002 (23.9)	1452 (16.5)	584 (8)	253 (19.1)	502 (19.8)	559 (14.1)	93652 (24.5)
**Q3 (%)**	72708 (17.9)	1825 (20.8)	1106 (15.2)	282 (21.3)	436 (17.2)	703 (17.7)	68356 (17.9)
**Q4 (%)**	63404 (15.6)	2268 (25.8)	2068 (28.4)	227 (17.1)	524 (20.7)	962 (24.3)	57355 (15)
**Q5 (%)**	51984 (12.8)	1901 (21.6)	3127 (43)	192 (14.5)	583 (23)	1173 (29.6)	45008 (11.8)
**Comorbidities**							
**Myocardial infarction (%)**	16389 (4)	580 (6.6)	152 (2.1)	15 (1.1)	75 (3)	137 (3.5)	15430 (4)
**Congestive heart failure (%)**	11282 (2.8)	267 (3)	178 (2.4)	7 (0.5)	36 (1.4)	89 (2.2)	10705 (2.8)
**Peripheral vascular disease (%)**	11643 (2.9)	242 (2.8)	140 (1.9)	17 (1.3)	54 (2.1)	75 (1.9)	11115 (2.9)
**Cerebrovascular disease (%)**	18968 (4.7)	347 (3.9)	324 (4.5)	26 (2)	92 (3.6)	157 (4)	18022 (4.7)
**Dementia (%)**	4299 (1.1)	80 (0.9)	87 (1.2)	5 (0.4)	16 (0.6)	24 (0.6)	4087 (1.1)
**Chronic pulmonary disease (%)**	77899 (19.2)	1761 (20)	1315 (18.1)	183 (13.8)	536 (21.1)	719 (18.1)	73385 (19.2)
**Rheumatologic disease (%)**	15635 (3.9)	373 (4.2)	305 (4.2)	24 (1.8)	92 (3.6)	128 (3.2)	14713 (3.9)
**Peptic ulcer disease (%)**	15830 (3.9)	381 (4.3)	334 (4.6)	60 (4.5)	94 (3.7)	191 (4.8)	14770 (3.9)
**Mild liver disease (%)**	9890 (2.4)	224 (2.5)	200 (2.7)	50 (3.8)	69 (2.7)	139 (3.5)	9208 (2.4)
**Diabetes (%)**	42222 (10.4)	2687 (30.6)	1693 (23.3)	161 (12.2)	301 (11.9)	817 (20.6)	36563 (9.6)

For peer review only

	All	Asian	Black	Chinese	Mixed	Other	White
**Hemiplegia (%)**	3278 (0.8)	80 (0.9)	95 (1.3)	6 (0.5)	20 (0.8)	32 (0.8)	3045 (0.8)
**Moderate or severe renal disease (%)**	27065 (6.7)	631 (7.2)	750 (10.3)	47 (3.5)	155 (6.1)	246 (6.2)	25236 (6.6)
**Cancer (%)**	55122 (13.6)	665 (7.6)	703 (9.7)	106 (8)	255 (10.1)	365 (9.2)	53028 (13.9)
**AIDS (%)**	533 (0.1)	9 (0.1)	41 (0.6)	<5 (0.2)	5 (0.2)	17 (0.4)	458 (0.1)

Occurrence of COVID-19 outcomes

Of the entire cohort, 18172 (4.5%) individuals tested positive, and a further 3285 (0.8%) tested positive following a previous negative result. 1490 individuals (6.9% of those who tested positive) were hospitalised following a positive result, and a further 2322 (0.6%) were directly hospitalised. Of those who were hospitalised, 662 individuals (17.4%) subsequently died within 28 days of a positive COVID test. Full descriptive statistics of the transitions between each COVID state are shown in Supplementary Table 1, stratified by ethnicity, socio-economic deprivation, sex and age.

Risks of testing positive, hospitalisation and death

Stratification of the cohort by ethnicity revealed differences between different ethnic groups in their risk of transitioning between different COVID states (Figure 3, Supplementary Table 2). Compared to White participants, Asian participants had an increased risk of testing positive (1.40 [1.30-1.51]) and direct hospitalisation (1.32 [1.04-1.67]), and a reduced risk of testing negative (0.92 [0.88-0.95]). Black participants had an increased risk of hospitalisation following a positive test (1.51 [1.14-2.01]) and direct hospitalisation (1.54 [1.22-1.94]). In contrast, Chinese participants had a reduced risk of testing negative (0.67 [0.59-0.76]) and testing positive (0.41 [0.29-0.58]). Participants with mixed or 'other' ethnicity did not have any notable differences in their risks of transitioning between any states compared with White participants. Once hospitalised, no ethnic group had increased risk of death compared to white participants.

Given the multistate model only accounts for the first positive or negative test an individual takes, Quasi-Poisson regression was used to compare differences in the amount of COVID testing between groups (Table 2). Black (1.15 [1.08-1.22]) and 'other ethnicity' (1.13 [1.03-1.23]) participants had an increased likelihood of testing for COVID-19, whereas in contrast Chinese participants had a reduced likelihood (0.70 [0.58-0.86]).

For peer review only - http://bmjopen.bmj.com/site/about/guidelines.xhtml

Table 2. Quasi-Poisson regression coefficients for number of tests taken, stratified by ethnicity, and shown at different levels of confounding. Fully adjusted includes adjustment for age, sex and socio-economic deprivation. Risk ratios are the exponential of the regression estimates, with the corresponding confidence intervals. Quasi-Poisson model results are shown due to overdispersion in the sample.

	**Quasi-Poisson**<br>**Risk ratio [95% CI]**
**Asian vs White**	
Unadjusted	1.10 [1.04,1.17]
Age-sex adjusted	1.14 [1.07,1.22]
Fully adjusted	1.04 [0.97,1.10]
**Black vs White**	
Unadjusted	1.27 [1.19,1.35]
Age-sex adjusted	1.35 [1.26,1.44]
Fully adjusted	1.15 [1.08,1.22]
**Chinese vs White**	
Unadjusted	0.64 [0.52,0.78]
Age-sex adjusted	0.68 [0.55,0.83]
Fully adjusted	0.70 [0.58,0.86]
**Mixed vs White**	
Unadjusted	0.98 [0.87,1.1]
Age-sex adjusted	1.05 [0.93,1.19]

For peer review only

For peer review only - http://bmjopen.bmj.com/site/about/guidelines.xhtml

Fully adjusted	0.98 [0.87,1.10]
**Other vs White**	
Unadjusted	1.15 [1.05,1.26]
Age-sex adjusted	1.22 [1.11,1.33]
Fully adjusted	1.13 [1.03,1.23]

[a] Fully adjusted for age, sex and socio-economic deprivation.

[b] [] <- 95% Confidence intervals

In addition, we extracted data concerning COVID-19 vaccine uptake among our cohort, covering both the first and second doses of the vaccine (Table 3). With regards to the first dose, all ethnic groups except Asian were less likely to have been vaccinated at the time of writing, compared to White participants. Asian and mixed ethnicity had similar chances than White participants to receive their second dose of the vaccine, whereas Black and Chinese participants were less likely to.

Table 3. Uptake risk of first and second doses of COVID-19 vaccines, stratified by ethnic background.

	First dose	Second dose
**Asian vs White**		
Unadjusted	0.82 [0.8,0.83]	0.86 [0.84,0.88]
Age-sex adjusted	0.99 [0.96,1.01]	1.08 [1.05,1.10]
Fully adjusted	0.99 [0.97,1.01]	1.09 [1.06,1.11]
**Black vs White**		

For peer review only - http://bmjopen.bmj.com/site/about/guidelines.xhtml

Unadjusted	0.52 [0.51,0.54]	0.69 [0.67,0.71]
Age-sex adjusted	0.61 [0.60,0.63]	0.79 [0.77,0.81]
Fully adjusted	0.63 [0.61,0.64]	0.81 [0.78,0.83]
**Chinese vs White**		
Unadjusted	0.65 [0.61,0.69]	0.74 [0.69,0.78]
Age-sex adjusted	0.78 [0.74,0.83]	0.90 [0.85,0.95]
Fully adjusted	0.79 [0.74,0.83]	0.90 [0.85,0.95]
**Mixed vs White**		
Unadjusted	0.66 [0.64,0.69]	0.75 [0.72,0.79]
Age-sex adjusted	0.85 [0.81,0.89]	1.03 [0.99,1.08]
Fully adjusted	0.85 [0.82,0.89]	1.04 [1.00,1.09]
**Other vs White**		
Unadjusted	0.62 [0.60,0.64]	0.8 [0.77,0.82]
Age-sex adjusted	0.74 [0.71,0.76]	1.01 [0.97,1.04]
Fully adjusted	0.76 [0.73,0.79]	1.02 [0.99,1.06]

[a] Fully adjusted for age, sex, socio-economic deprivation and comorbidities.

[b] [] <- 95% Confidence intervals

For peer review only - http://bmjopen.bmj.com/site/about/guidelines.xhtml

Discussion

Statement of principle findings

Our study of 405,541 participants of the UK Biobank demonstrates significant differences in risks of various COVID-related outcomes between different ethnic groups. Compared to White participants, we found that Black and Asian participants have clearly increased risks of testing positive for COVID-19 and of being hospitalised for the disease. Whilst our data suggests an increased risk of death following hospitalisation, the high uncertainty for this transition means that higher risk cannot be reliably asserted.

Both Asian and Black participants had a higher likelihood of being tested for COVID-19, whereas Chinese participants were less likely to be, compared to White participants. In terms of vaccine uptake, our study shows White and Asian participants had a higher likelihood of receiving their first vaccine dose compared to other ethnic groups. Compared to White participants, Asian and mixed ethnicity participants were more likely to receive their second vaccine dose, whereas Black and Chinese participants were less likely to.

Strengths and weaknesses of the study

Our study has several strengths. Firstly, it includes a large number of participants from an established database, allowing extraction of both baseline characteristics as well as linked information related to COVID-19 testing and hospitalisation, helping to avoid issues around the self-reporting of COVID-related events and outcomes. Secondly, by choosing a start date for follow-up to coincide with the initiation of wider population testing in the community for COVID-19, we address issues of collider bias intrinsic to similar studies that focussed on the early first wave of the pandemic, when testing was more limited. Thirdly, our follow-up window lasts for 18 months, allowing the inclusion of higher numbers of COVID-19 cases, as well as providing an up-to-date analysis now that the UK has experienced multiple waves of the pandemic.

However, our study also has some weaknesses. Comparative studies have shown that the UK Biobank is not optimally representative of the wider population, with UK Biobank participants tending to be older, more female, healthier, and less socioeconomically deprived, as we show in our description of our cohort's baseline characteristics, which limits the generalisability of our findings. [37] Our approach to confounders helps to address some of these issues. but we still have a very selected population Although we adjust for socio-economic status as a confounder, the deprivation index used is based on territorial units and not on individual characteristics, so it does not contain information pertaining to an individual. For example, we don't measure occupation, an important driver of COVID, and most of our population is in retirement age.

For peer review only - http://bmjopen.bmj.com/site/about/guidelines.xhtml

An important consideration is the potential confounding effect that differential vaccine uptake, illustrated in our results above, may have on our primary outcomes in the multi-state model. Given the established effectiveness of vaccines in preventing severe disease, the vaccinated status of individuals in our study may have affected their risk of progressing through our multi-state model. By design, our study could not include individuals who developed COVID-19 but did not test for it, or individuals who died with undiagnosed COVID-19. Also, the number of outcomes other than negative or positive tests in some ethnicity categories is quite low, so those should be interpreted with caution. We did not evaluate interactions that may be relevant, such as gender, age and SES with ethnicity, due to low number of outcomes. We also recognise that the UK Biobank's initial categorisation of ethnicity, used by necessity in this study, differs from the current UK Government Statistical Service ethnicity harmonised standard. Differences in linkage to tests, with some laboratories not reporting negative tests, and availability and differential use and reporting of home testing could also have biased the results.

Strengths and weaknesses in relation to other studies, discussing important differences in results

Our study has several strengths in comparison to previous studies examining COVID outcomes using the UK Biobank. Our longer observation period and use of start dates in conjunction with wider population testing helps address intrinsic biases, and our multistate approach allows a more detailed assessment of different groups risks of specific outcomes than previously reported.[38]

This approach has allowed us to confirm several findings of previous studies, mainly conducted in a timeframe where testing was only performed on hospital, namely that Black and Asian individuals have a higher risk of testing positive for COVID-19 (indicating severe disease as most where hospitalised). [2,11,23,39,40] We found that once more wide availability of tests started, we still find an increased risk of testing positive in hospital for Black and Asian people, and increased risk of hospitalisation once testing positive for Blacks, but a risk of testing positive outside hospital and testing negative similar to Whites. Our results also show increased testing rates among Black and Asian participants also supports studies undertaken in the UK using other data sources and methods, such as large scale primary care data[41].

[42][23]We found a test positivity rate similar to the general population but the case fatality rate seems to be higher than those of the general population for the same period. These differences are probably related to UK Biobank being composed of people of relatively older groups than the UK population.[42] We also found an increased mortality for those hospitalised with COVID-19 amongst Black, Asian and Chinese ethnic groups, although with high uncertainty due to low numbers. This is in line with previous studies, [9,18] but probably with a slightly lower due to those studies studying mortality in general population, and our lethality among the hospitalised.

Meaning of the study: possible explanations and implications for clinicians and policymakers

For peer review only - http://bmjopen.bmj.com/site/about/guidelines.xhtml

The underlying causes of the inequalities demonstrated by our study are likely to be multifactorial, complex, and varied.

Ethnic minority groups are disproportionately represented in lower socio-economic groups, and deprivation is an established driver of poorer health outcomes. In our data we could not disentangle deprivation effects from other related effects, like occupation, other household inhabitants occupation, or living conditions. Data from the US has suggested that ethnic hurminority groups are disproportionately represented in frontline roles which may increase exposure to the virus.[43] Similarly, in the UK, data from the Office for National Statistics has shown that 'key-workers' are more likely than average to be from an ethnic minority group.[44] There is also evidence suggesting that BAME groups in the UK are more likely to be employed in public-facing jobs where physical distancing is difficult (e.g., transport). [45,46] Finally, there is evidence that ethnic minorities are more likely to live in overcrowded households, which represents a risk factor for COVID-19 diagnosis and severity.

In addition, structural and cultural racism and discrimination are also likely to be important contributors to the adverse outcomes experienced by ethnic minority groups during the pandemic.[47] It is established that ethnic disparities exist in a wide range of other healthcare contexts, such as maternal deaths[48] and sectioning under the Mental Health Act.[49] This has been thought to be multifactorial. Practical aspects as transport availability and caring duties could have played a role. [50] Another possibly more important factor is racial discrimination in medical settings and other institutional barriers, as inadequate communication, and culturally insensitive care in the NHS. Studies have shown that implicit biases not only affect communication with patients, but also their experiences and outcomes of their clinical care.[51,52] This leads to lower trust in medical systems, and a higher susceptibility to targeted misinformation. [50,53,54] These inbuilt systemic biases towards ethnic minorities can be directly contributing to poorer outcomes after COVID-19 infection, due to delays in care, to the lower vaccination rate seen in the analyses, and to differential access to home testing and testing reporting.

Unanswered questions and future research

Further research using the datasets from this study, for example, could investigate availability of testing and vaccination by territorial region, which is a source of systemic bias. More research into the underlying causes of ethnic disparities in pandemic-related outcomes will be important, especially when designing practical, effective and appropriate public health policy responses to future pandemics.

For peer review only - http://bmjopen.bmj.com/site/about/guidelines.xhtml

Competing interests

Prof. Prieto-Alhambra's research group has received grant support from Amgen, Chesi-Taylor, Novartis, and UCB Biopharma. His department has received advisory or consultancy fees from Amgen, Astellas, AstraZeneca, Johnson, and Johnson, and UCB Biopharma and fees for speaker services from Amgen and UCB Biopharma. Janssen, on behalf of IMI-funded EHDEN and EMIF consortiums, and Synapse Management Partners have supported training programmes organised by DPA's department and open for external participants organized by his department outside submitted work.

Funding statement

The research was supported by the National Institute for Health Research (NIHR) Oxford Biomedical Research Centre (BRC). DPA is funded through a NIHR Senior Research Fellowship (Grant number SRF-2018-11-ST2-004). The views expressed in this publication are those of the author(s) and not necessarily those of the NHS, the National Institute for Health Research, or the Department of Health.

Data sharing statement

UK Biobank data are available under specific conditions directly from the UK Biobank platform: https://www.ukbiobank.ac.uk

Ethics approval

The North West Multi-center Research Ethics Committee (MREC) approved the collection and use of UK Biobank data (16/NW/0274). UKBiobank approved the application used to access the data (Application 46228) on 17/07/2019 and its access to COVID-19 data on 17/04/2020.

Statement of transparency

The lead author affirms that this manuscript is an honest, accurate, and transparent account of the study being reported; that no important aspects of the study have been omitted; and that any discrepancies from the study as planned (and, if relevant, registered) have been explained. Tomás Urdiales and Albert Prats-Uribe directly conducted the analyses and had access to the data.

Contributorship statement

DPA and CP conceived and designed the study. TU, APU and MC acquired and performed analyses of the data, and all authors were involved in interpretation of the findings. FD, TU and APU drafted the work, and all authors were involved in critical revisions of the subsequent drafts. All authors gave final approval of the version to be published, and agree to be accountable for all aspects of the work.

For peer review only - http://bmjopen.bmj.com/site/about/guidelines.xhtml

Acknowledgemetns

This research has been conducted using the UK Biobank Resource under Application Number 46228. This work uses data provided by patients and collected by the NHS as part of their care and support. This data is copyrighted, © 2022, NHS England. Re-used with the permission of the NHS England and UK Biobank. All rights reserved.

For peer review only

References

1 Dong E, Du H, Gardner L. An interactive web-based dashboard to track COVID-19 in real time. *Lancet Infect Dis* 2020;**20**:533–4. doi:10.1016/S1473-3099(20)30120-1

2 Sze S, Pan D, Nevill CR, *et al.* Ethnicity and clinical outcomes in COVID-19: A systematic review and meta-analysis. *EClinicalMedicine* 2020;**29–30**:100630. doi:10.1016/j.eclinm.2020.100630

3 Apea VJ, Wan YI, Dhairyawan R, *et al.* Ethnicity and outcomes in patients hospitalised with COVID-19 infection in East London: An observational cohort study. *BMJ Open* 2021;**11**:1–11. doi:10.1136/bmjopen-2020-042140

4 Sapey E, Gallier S, Mainey C, *et al.* Ethnicity and risk of death in patients hospitalised for COVID-19 infection in the UK: An observational cohort study in an urban catchment area. *BMJ Open Respir Res* 2020;**7**:1–11. doi:10.1136/bmjresp-2020-000644

5 Griffith GJ, Morris TT, Tudball MJ, *et al.* Collider bias undermines our understanding of COVID-19 disease risk and severity. *Nat Commun* 2020;**11**:1–12. doi:10.1038/s41467-020-19478-2

6 Sudlow C, Gallacher J, Allen N, *et al.* UK Biobank: An Open Access Resource for Identifying the Causes of a Wide Range of Complex Diseases of Middle and Old Age. *PLoS Med* 2015;**12**:1–10. doi:10.1371/journal.pmed.1001779

7 Armstrong J, Rudkin JK, Allen N, *et al.* Dynamic linkage of covid-19 test results between public health england's second generation surveillance system and uk biobank. *Microb Genom* 2020;**6**:1–9. doi:10.1099/mgen.0.000397

8 Atkins JL, Masoli JAH, Delgado J, *et al.* Preexisting Comorbidities Predicting COVID-19 and Mortality in the UK Biobank Community Cohort. *Journals of Gerontology - Series A Biological Sciences and Medical Sciences* 2020;**75**:2224–30. doi:10.1093/gerona/glaa183

9 Batty GD, Gaye B, Gale CR, *et al.* Explaining Ethnic Differentials in COVID-19 Mortality: A Cohort Study. *Am J Epidemiol* 2022;**191**:275–81. doi:10.1093/aje/kwab237

10 Patel AP, Paranjpe MD, Kathiresan NP, *et al.* Race, socioeconomic deprivation, and hospitalization for COVID-19 in English participants of a national biobank. *Int J Equity Health* 2020;**19**:1–4. doi:10.1186/s12939-020-01227-y

11 Raisi-Estabragh Z, McCracken C, Bethell MS, *et al.* Greater risk of severe COVID-19 in black, asian and minority ethnic populations is not explained by cardiometabolic, socioeconomic or behavioural factors, or by 25(OH)-vitamin D status: Study of 1326 cases from the UK biobank. *Journal of Public Health (United Kingdom)* 2020;**42**:451–60. doi:10.1093/pubmed/fdaa095

12 Razieh C, Zaccardi F, Gillies CL, *et al.* Ethnic minorities and COVID-19: examining whether excess risk is mediated through deprivation. *Eur J Public Health* 2021;**31**:630–4. doi:10.1093/eurpub/ckab041

13 Sattar N, Ho FK, Gill JM, *et al.* BMI and future risk for COVID-19 infection and death across sex, age and ethnicity: Preliminary findings from UK biobank. *Diabetes and Metabolic Syndrome: Clinical Research and Reviews* 2020;**14**:1149–51. doi:10.1016/j.dsx.2020.06.060

For peer review only - http://bmjopen.bmj.com/site/about/guidelines.xhtml

14 Woolford SJ, D'Angelo S, Curtis EM, *et al.* COVID-19 and associations with frailty and multimorbidity: a prospective analysis of UK Biobank participants. *Aging Clin Exp Res* 2020;**32**:1897–905. doi:10.1007/S40520-020-01653-6

15 Woodward M, Peters SAE, Harris K. Social deprivation as a risk factor for COVID-19 mortality among women and men in the UK Biobank: nature of risk and context suggests that social interventions are essential to mitigate the effects of future pandemics. *J Epidemiol Community Health (1978)* 2021;**75**. doi:10.1136/JECH-2020-215810

16 Chadeau-Hyam M, Bodinier B, Elliott J, *et al.* Risk factors for positive and negative COVID-19 tests: A cautious and in-depth analysis of UK biobank data. *Int J Epidemiol* 2020;**49**:1454–67. doi:10.1093/ije/dyaa134

17 Chudasama Y V., Zaccardi F, Gillies CL, *et al.* Patterns of multimorbidity and risk of severe SARS-CoV-2 infection: an observational study in the U.K. *BMC Infect Dis* 2021;**21**:1–12. doi:10.1186/s12879-021-06600-y

18 Elliott J, Bodinier B, Whitaker M, *et al.* COVID-19 mortality in the UK Biobank cohort: revisiting and evaluating risk factors. *Eur J Epidemiol* 2021;**36**:299–309. doi:10.1007/s10654-021-00722-y

19 Ho FK, Celis-Morales CA, Gray SR, *et al.* Modifiable and non-modifiable risk factors for COVID-19, and comparison to risk factors for influenza and pneumonia: Results from a UK Biobank prospective cohort study. *BMJ Open* 2020;**10**. doi:10.1136/bmjopen-2020-040402

20 Kolin DA, Kulm S, Christos PJ, *et al.* Clinical, regional, and genetic characteristics of Covid-19 patients from UK Biobank. *PLoS One* 2020;**15**:1–13. doi:10.1371/journal.pone.0241264

21 Lassale C, Gaye B, Hamer M, *et al.* Ethnic disparities in hospitalisation for COVID-19 in England: The role of socioeconomic factors, mental health, and inflammatory and pro-inflammatory factors in a community-based cohort study. *Brain Behav Immun* 2020;**88**:44–9. doi:10.1016/j.bbi.2020.05.074

22 McQueenie R, Foster HME, Jani BD, *et al.* Multimorbidity, polypharmacy, and COVID-19 infection within the UK Biobank cohort. *PLoS One* 2020;**15**:1–15. doi:10.1371/journal.pone.0238091

23 Niedzwiedz CL, O'Donnell CA, Jani BD, *et al.* Ethnic and socioeconomic differences in SARS-CoV-2 infection: Prospective cohort study using UK Biobank. *BMC Med* 2020;**18**:1–14. doi:10.1186/s12916-020-01640-8

24 UK Biobank. UK Biobank Mortality data: linkage to death registries. Published Online First: 2020.http://www.ukbiobank.ac.uk/ (accessed 2 Apr 2023).

25 Herbert A, Wijlaars L, Zylbersztejn A, *et al.* Data Resource Profile: Hospital Episode Statistics Admitted Patient Care (HES APC). *Int J Epidemiol* 2017;**46**:1093–1093i. doi:10.1093/IJE/DYX015

26 Meira-Machado LF, de Uña-Álvarez J, Cadarso-Suárez C, *et al.* Multi-state models for the analysis of time-to-event data. *Stat Methods Med Res* 2009;**18**:195–222. doi:10.1177/0962280208092301

27 Burn E, Tebé C, Fernandez-Bertolin S, *et al.* The natural history of symptomatic COVID-19 during the first wave in Catalonia. *Nat Commun* 2021;**12**. doi:10.1038/S41467-021-21100-Y

28 Flanagin A, Frey T, Christiansen SL. Updated Guidance on the Reporting of Race and Ethnicity in Medical and Science Journals. *JAMA* 2021;**326**:621–7. doi:10.1001/JAMA.2021.13304

For peer review only - http://bmjopen.bmj.com/site/about/guidelines.xhtml

29 English indices of deprivation 2019 - GOV.UK. https://www.gov.uk/government/statistics/english-indices-of-deprivation-2019 (accessed 2 Apr 2023).
30 Sundararajan V, Henderson T, Perry C, *et al.* New ICD-10 version of the Charlson comorbidity index predicted in-hospital mortality. *J Clin Epidemiol* 2004;**57**:1288–94. doi:10.1016/J.JCLINEPI.2004.03.012
31 Xie J, Prats-Uribe A, Feng Q, *et al.* Clinical and Genetic Risk Factors for Acute Incident Venous Thromboembolism in Ambulatory Patients With COVID-19. *JAMA Intern Med* 2022;**182**:1063–70. doi:10.1001/JAMAINTERNMED.2022.3858
32 Kaplan EL, Meier P. Nonparametric Estimation from Incomplete Observations. *J Am Stat Assoc* 1958;**53**:457. doi:10.2307/2281868
33 Wickham H, Averick M, Bryan J, *et al.* Wickham et al., (2019). Welcome to the tidyverse. Journal of Open Source Software, 4(43), 1686. https://doi.org/10.21105/joss.01686
34 de Wreede LC, Fiocco M, Putter H. mstate: An R Package for the Analysis of Competing Risks and Multi-State Models. *J Stat Softw* 2011;**38**:1–30. doi:10.18637/JSS.V038.I07
35 Therneau MT. Therneau T (2020). A Package for Survival Analysis in R. R package version 3.2-3. 2020;:1–172.Therneau T (2020). A Package for Survival Analysis in R. R package version 3.2-3
36 kableExtra: Construct Complex Table with 'kable' and Pipe Syntax version 1.3.4 from CRAN. https://rdrr.io/cran/kableExtra/ (accessed 2 Apr 2023).
37 Fry A, Littlejohns TJ, Sudlow C, *et al.* Comparison of Sociodemographic and Health-Related Characteristics of UK Biobank Participants With Those of the General Population. *Am J Epidemiol* 2017;**186**:1026–34. doi:10.1093/AJE/KWX246
38 Hastie CE, Mackay DF, Ho F, *et al.* Vitamin D concentrations and COVID-19 infection in UK Biobank. *Diabetes and Metabolic Syndrome: Clinical Research and Reviews* 2020;**14**:561–5. doi:10.1016/j.dsx.2020.04.050
39 Agyemang C, Richters A, Jolani S, *et al.* Ethnic minority status as social determinant for COVID-19 infection, hospitalisation, severity, ICU admission and deaths in the early phase of the pandemic: A meta-analysis. *BMJ Glob Health* 2021;**6**:1–14. doi:10.1136/bmjgh-2021-007433
40 Magesh S, John D, Li WT, *et al.* Disparities in COVID-19 Outcomes by Race, Ethnicity, and Socioeconomic Status: A Systematic-Review and Meta-analysis. *JAMA Netw Open* 2021;**4**:1–16. doi:10.1001/jamanetworkopen.2021.34147
41 Mathur R, Rentsch CT, Morton CE, *et al.* Ethnic differences in SARS-CoV-2 infection and COVID-19-related hospitalisation, intensive care unit admission, and death in 17 million adults in England: an observational cohort study using the OpenSAFELY platform. *The Lancet* 2021;**397**:1711–24. doi:10.1016/S0140-6736(21)00634-6
42 England Summary | Coronavirus (COVID-19) in the UK. UK Government. 2023.https://coronavirus.data.gov.uk/ (accessed 2 Apr 2023).
43 Goldman N, Pebley AR, Lee K, *et al.* Racial and ethnic differentials in COVID-19-related job exposures by occupational standing in the US. *PLoS One* 2021;**16**:1–17. doi:10.1371/journal.pone.0256085
44 https://commonslibrary.parliament.uk/coronavirus-which-key-workers-are-most-at-risk/.

For peer review only

For peer review only - http://bmjopen.bmj.com/site/about/guidelines.xhtml

45 Khunti K, Platt L, Routen A, *et al.* Covid-19 and ethnic minorities: an urgent agenda for overdue action The government's report falls seriously short on commitment. Published Online First: 2020. doi:10.1136/bmj.m2503
46 Magesh S, John D, Li WT, *et al.* Disparities in COVID-19 Outcomes by Race, Ethnicity, and Socioeconomic Status: A Systematic Review and Meta-analysis. *JAMA Netw Open* 2021;**4**:e2134147–e2134147. doi:10.1001/JAMANETWORKOPEN.2021.34147
47 Razai MS, Kankam HKN, Majeed A, *et al.* Mitigating ethnic disparities in covid-19 and beyond. *The BMJ* 2021;**372**:1–5. doi:10.1136/bmj.m4921
48 Knight M, Bunch K, Tuffnell D, *et al.* MBRRACE-UK Saving Lives, Improving Mothers' Care – Lessons learned to inform maternity care from the UK and Ireland Confidential Enquiries into Maternal Deaths and Morbidity 2017-19. Published Online First: 2011.https://www.npeu.ox.ac.uk/mbrrace-uk/reports
49 GOV.UK. Modernising the Mental Health Act - Final report of the Independent Review of the Mental Health Act 1983. 2018;:1–322.www.gov.uk/dhsc
50 Yong A, Germain S. Ethnic minority and migrant women's struggles in accessing healthcare during COVID-19: an intersectional analysis. *https://doi.org/101080/1479758520212012090* 2022;**26**:65–82. doi:10.1080/14797585.2021.2012090
51 Cooper LA, Roter DL, Carson KA, *et al.* The associations of clinicians' implicit attitudes about race with medical visit communication and patient ratings of interpersonal care. *Am J Public Health* 2012;**102**:979–87. doi:10.2105/AJPH.2011.300558
52 Van Ryn M, Burgess DJ, Dovidio JF, *et al.* The impact of racism on clinician cognition, behavior, and clinical decision making. *Du Bois Review* 2011;**8**:199–218. doi:10.1017/S1742058X11000191
53 Gaughan CH, Razieh C, Khunti K, *et al.* COVID-19 vaccination uptake amongst ethnic minority communities in England: a linked study exploring the drivers of differential vaccination rates. *J Public Health (Oxf)* 2023;**45**:e65–74. doi:10.1093/PUBMED/FDAB400
54 Paul E, Fancourt D, Razai M. Racial discrimination, low trust in the health system and COVID-19 vaccine uptake: a longitudinal observational study of 633 UK adults from ethnic minority groups. *J R Soc Med* 2022;**115**:439–47. doi:10.1177/01410768221095241

For peer review only

For peer review only - http://bmjopen.bmj.com/site/about/guidelines.xhtml

Figure legend

Figure 1. Overview of the multistate model used in this study with the four "COVID-19 states" and the possible transitions.

Figure 2. Flowchart outlining stepwise exclusions of participants

Figure 3. Forest plots of ethnicity-stratified hazard ratios, fully adjusted for age, sex and socioeconomic deprivation. The scale used for visualisation is linear but differs between each transition. The three dots reflect the x=1 axis.

For peer review only

For peer review only - http://bmjopen.bmj.com/site/about/guidelines.xhtml

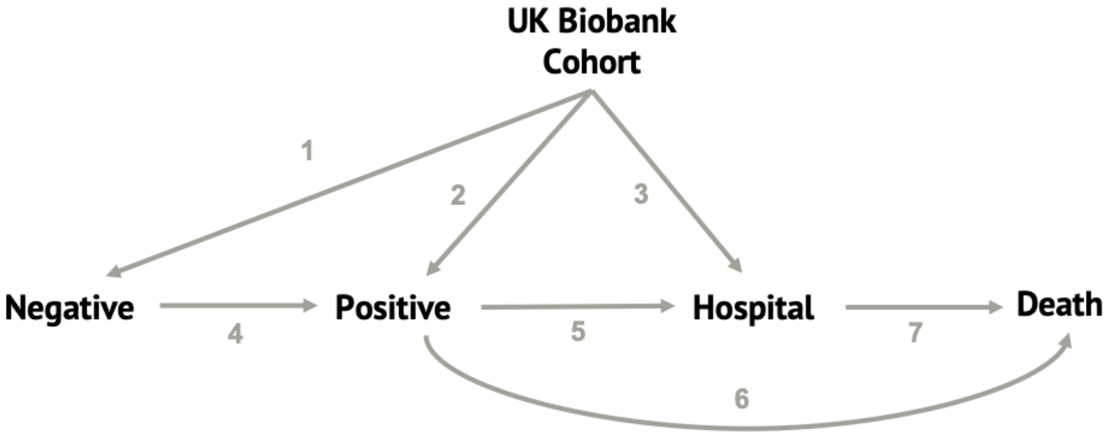

For peer review only

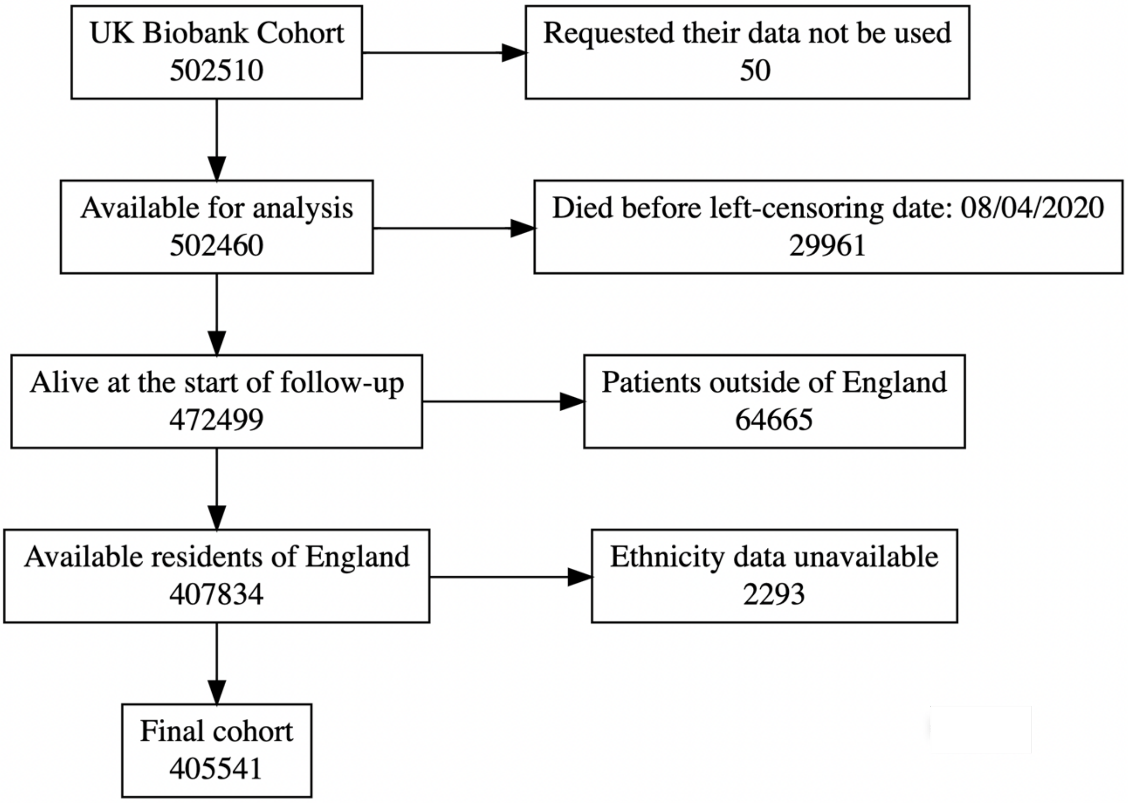
UK Biobank Cohort
502510
Requested their data not be used
50
Available for analysis
502460
Died before left-censoring date: 08/04/2020
29961
Alive at the start of follow-up
472499
Patients outside of England
64665
Available residents of England
407834
Ethnicity data unavailable
2293
Final cohort
405541

eview only

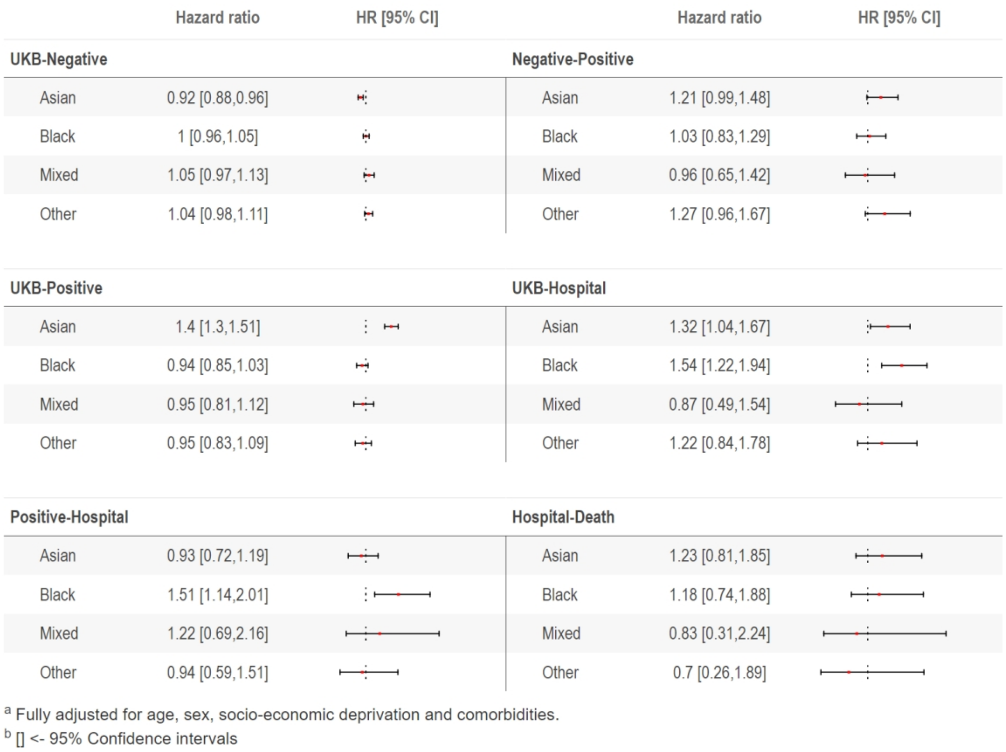

Forest plots of ethnicity-stratified hazard ratios, fully adjusted for age, sex, socioeconomic deprivation and comorbidities. The scale used for visualisation is linear but differs between each transition. The three dots reflect the x=1 axis.

854x653mm (47 x 47 DPI)

Supplementary Figure 1. Radar charts for socio-economic deprivation quintile population distribution, stratified by ethnicity. The distribution of each ethnic group is shown by the coloured pentagons, in comparison to the whole cohort distribution in dark grey beneath.

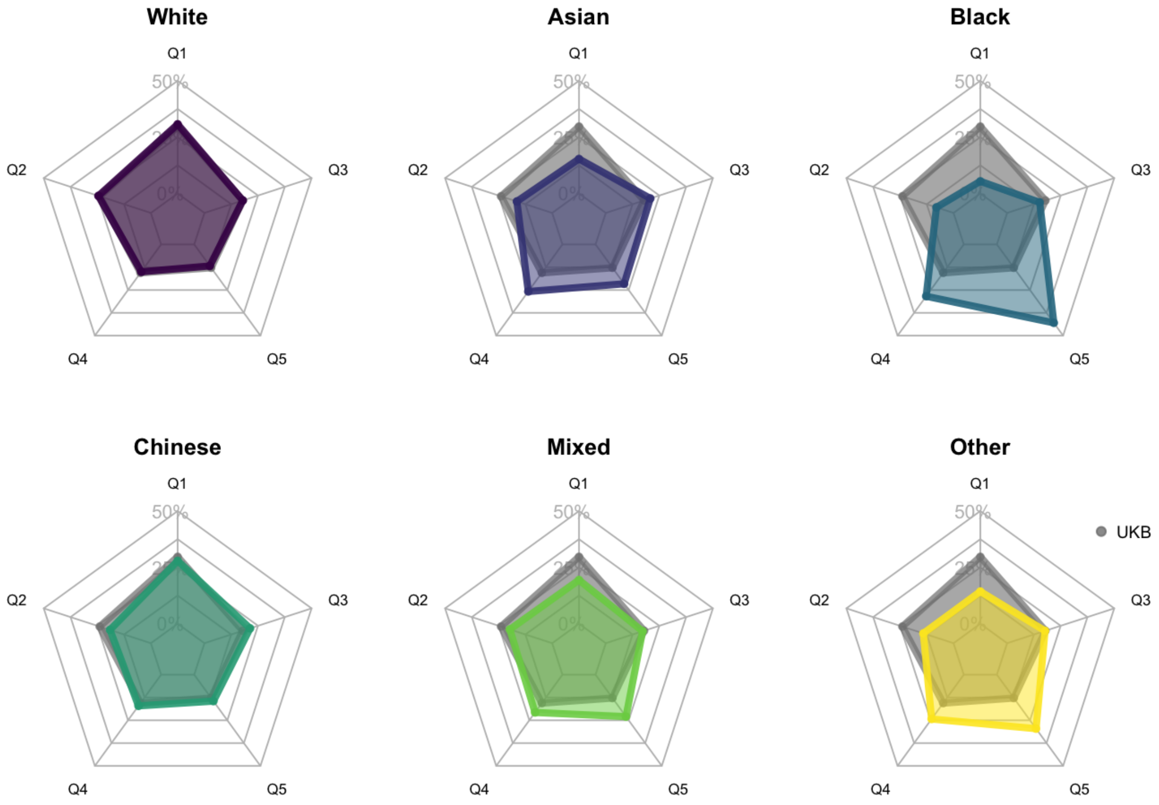

Supplementary Table 1. COVID outcome event numbers. Stratified by ethnicity, socioeconomic deprivation quintile, sex and age. All percentages (in brackets) are calculated as the fraction of individuals who underwent a particular transition from the total who were in the states preceding it (and thus susceptible to transitioning). Age is divided into those younger and older than the median age 70.8.

	Total	UKB-Negative	Negative-Positive	UKB-Positive	Positive-Hospital	Positive-Death	UKB-Hospital	Hospital-Death
All (%)	405541	111614 (27.5)	3285 (2.9)	18172 (4.5)	1490 (6.9)	129 (0.6)	2322 (0.6)	662 (17.4)
**Ethnicity**								
Asian (%)	8791	2132 (24.3)	100 (4.7)	714 (8.1)	68 (8.4)	4 (0.5)	75 (0.9)	25 (17.5)
Black (%)	7275	1883 (25.9)	84 (4.5)	450 (6.2)	52 (9.7)	3 (0.6)	82 (1.1)	19 (14.2)
Chinese (%)	1324	238 (18)	4 (1.7)	31 (2.3)	3 (8.6)	0 (0)	7 (0.5)	2 (20)
Mixed (%)	2537	688 (27.1)	25 (3.6)	146 (5.8)	12 (7)	0 (0)	12 (0.5)	4 (16.7)
Other (%)	3964	1068 (26.9)	53 (5)	226 (5.7)	18 (6.5)	2 (0.7)	28 (0.7)	4 (8.7)
White (%)	381650	105605 (27.7)	3019 (2.9)	16605 (4.4)	1337 (6.8)	120 (0.6)	2118 (0.6)	608 (17.6)
**Socio-economic deprivation**								
Q1 (%)	120443	34121 (28.3)	790 (2.3)	4112 (3.4)	290 (5.9)	34 (0.7)	478 (0.4)	127 (16.5)
Q2 (%)	97002	26912 (27.7)	741 (2.8)	4006 (4.1)	258 (5.4)	20 (0.4)	431 (0.4)	95 (13.8)
Q3 (%)	72708	19607 (27)	585 (3)	3454 (4.8)	286 (7.1)	18 (0.4)	391 (0.5)	132 (19.5)
Q4 (%)	63404	17235 (27.2)	615 (3.6)	3363 (5.3)	294 (7.4)	30 (0.8)	461 (0.7)	138 (18.3)
Q5 (%)	51984	13739 (26.4)	554 (4)	3237 (6.2)	362 (9.5)	27 (0.7)	561 (1.1)	170 (18.4)
**Sex**								

	Total	UKB-Negative	Negative-Positive	UKB-Positive	Positive-Hospital	Positive-Death	UKB-Hospital	Hospital-Death
Male (%)	181413	51172 (28.2)	1512 (3)	8365 (4.6)	829 (8.4)	72 (0.7)	1281 (0.7)	429 (20.3)
Female (%)	224128	60442 (27)	1773 (2.9)	9807 (4.4)	661 (5.7)	57 (0.5)	1041 (0.5)	233 (13.7)
**Age**								
Younger than 70 (%)	201131	51280 (12.6)	1970 (1.8)	12598 (3.1)	729 (3.4)	16 (0.1)	725 (0.2)	129 (3.4)
Older than 70 (%)	204410	60334 (14.9)	1315 (1.2)	5574 (1.4)	761 (3.5)	113 (0.5)	1597 (0.4)	533 (14)

For peer review only

Supplementary Table 2. Hazard ratio coefficients stratified by ethnicity, at different levels of confounding. Fully adjusted for age, sex, socioeconomic deprivation and comorbidities.

	UKB-Negative	Negative-Positive	UKB-Positive	Positive-Hospital	UKB-Hospital	Hospital-Death
**Asian vs White**						
Unadjusted	0.9 [0.86,0.93]	1.54 [1.26,1.88]	1.91 [1.77,2.06]	1.05 [0.82,1.34]	1.56 [1.24,1.97]	0.93 [0.62,1.39]
Age-sex adjusted	0.94 [0.9,0.98]	1.3 [1.07,1.59]	1.55 [1.44,1.67]	1.26 [0.99,1.61]	1.89 [1.5,2.39]	1.34 [0.9,2.01]
Fully adjusted	0.92 [0.88,0.96]	1.21 [0.99,1.48]	1.4 [1.3,1.51]	0.93 [0.72,1.19]	1.32 [1.04,1.67]	1.23 [0.81,1.85]
**Black vs White**						
Unadjusted	0.95 [0.91,1]	1.48 [1.2,1.84]	1.44 [1.31,1.58]	1.44 [1.09,1.9]	2 [1.6,2.51]	0.82 [0.52,1.3]
Age-sex adjusted	1.02 [0.98,1.07]	1.17 [0.94,1.45]	1.11 [1.01,1.22]	1.96 [1.48,2.59]	2.79 [2.22,3.49]	1.25 [0.79,1.98]
Fully adjusted	1 [0.96,1.05]	1.03 [0.83,1.29]	0.94 [0.85,1.03]	1.51 [1.14,2.01]	1.54 [1.22,1.94]	1.18 [0.74,1.88]
**Chinese vs White**						
Unadjusted	0.6 [0.53,0.69]	0.61 [0.23,1.62]	0.5 [0.35,0.71]	1.28 [0.41,3.99]	0.91 [0.43,1.91]	0.77 [0.19,3.1]
Age-sex adjusted	0.64 [0.57,0.73]	0.51 [0.19,1.36]	0.41 [0.29,0.58]	1.9 [0.61,5.92]	1.24 [0.59,2.61]	1.1 [0.27,4.4]
Fully adjusted	0.67 [0.59,0.76]	0.5 [0.19,1.33]	0.41 [0.29,0.58]	1.78 [0.57,5.56]	1.32 [0.63,2.77]	1.21 [0.3,4.87]
**Mixed vs White**						
Unadjusted	0.99 [0.92,1.06]	1.29 [0.87,1.91]	1.33 [1.13,1.57]	1 [0.57,1.76]	0.87 [0.49,1.53]	0.85 [0.32,2.27]

	UKB-Negative	Negative-Positive	UKB-Positive	Positive-Hospital	UKB-Hospital	Hospital-Death
Age-sex adjusted	1.07 [0.99,1.15]	1 [0.68,1.49]	1.02 [0.87,1.2]	1.41 [0.8,2.5]	1.24 [0.7,2.18]	1.03 [0.39,2.77]
Fully adjusted	1.05 [0.97,1.13]	0.96 [0.65,1.42]	0.95 [0.81,1.12]	1.22 [0.69,2.16]	0.87 [0.49,1.54]	0.83 [0.31,2.24]
**Other vs White**						
Unadjusted	0.98 [0.93,1.04]	1.76 [1.34,2.31]	1.32 [1.16,1.5]	0.84 [0.52,1.33]	1.3 [0.89,1.88]	0.51 [0.19,1.36]
Age-sex adjusted	1.04 [0.98,1.11]	1.41 [1.07,1.85]	1.06 [0.93,1.21]	1.08 [0.68,1.72]	1.72 [1.18,2.49]	0.76 [0.28,2.02]
Fully adjusted	1.04 [0.98,1.11]	1.27 [0.96,1.67]	0.95 [0.83,1.09]	0.94 [0.59,1.51]	1.22 [0.84,1.78]	0.7 [0.26,1.89]

[a] Fully adjusted for age, sex, socio-economic deprivation and comorbidities.

[b] [] <- 95% Confidence intervals

only

For peer review only

The RECORD statement – checklist of items, extended from the STROBE statement, that should be reported in observational studies using routinely collected health data.

	Item No.	STROBE items	Location in manuscript where items are reported	RECORD items	Location in manuscript where items are reported
**Title and abstract**					
	1	(a) Indicate the study's design with a commonly used term in the title or the abstract (b) Provide in the abstract an informative and balanced summary of what was done and what was found	Abstract (page 1)	RECORD 1.1: The type of data used should be specified in the title or abstract. When possible, the name of the databases used should be included.<br><br>RECORD 1.2: If applicable, the geographic region and timeframe within which the study took place should be reported in the title or abstract.<br><br>RECORD 1.3: If linkage between databases was conducted for the study, this should be clearly stated in the title or abstract.	Abstract (page 1)<br><br>Abstract (page 1)
**Introduction**					
Background rationale	2	Explain the scientific background and rationale for the investigation being reported	Introduction (page 3)		
Objectives	3	State specific objectives, including any prespecified hypotheses	Introduction (page 3)		
**Methods**					
Study Design	4	Present key elements of study design early in the paper	Methods (page 3-5)		
Setting	5	Describe the setting, locations, and relevant dates, including periods of recruitment, exposure, follow-up, and data collection	Methods (page 3-5)		

For peer review only

Participants	6	*(a) Cohort study* - Give the eligibility criteria, and the sources and methods of selection of participants. Describe methods of follow-up<br>*Case-control study* - Give the eligibility criteria, and the sources and methods of case ascertainment and control selection. Give the rationale for the choice of cases and controls<br>*Cross-sectional study* - Give the eligibility criteria, and the sources and methods of selection of participants<br><br>*(b) Cohort study* - For matched studies, give matching criteria and number of exposed and unexposed<br>*Case-control study* - For matched studies, give matching criteria and the number of controls per case	Methods (page 3-5)	RECORD 6.1: The methods of study population selection (such as codes or algorithms used to identify subjects) should be listed in detail. If this is not possible, an explanation should be provided.<br><br>RECORD 6.2: Any validation studies of the codes or algorithms used to select the population should be referenced. If validation was conducted for this study and not published elsewhere, detailed methods and results should be provided.<br><br>RECORD 6.3: If the study involved linkage of databases, consider use of a flow diagram or other graphical display to demonstrate the data linkage process, including the number of individuals with linked data at each stage.	Methods (page 3-5)
Variables	7	Clearly define all outcomes, exposures, predictors, potential confounders, and effect modifiers. Give diagnostic criteria, if applicable.	Methods (page 3-5)	RECORD 7.1: A complete list of codes and algorithms used to classify exposures, outcomes, confounders, and effect modifiers should be provided. If these cannot be reported, an explanation should be provided.	Methods (page 3-5)
Data sources/ measurement	8	For each variable of interest, give sources of data and details of methods of assessment (measurement).<br>Describe comparability of assessment methods if there is more than one group	Methods (page 3-5)		

For peer review only

Bias	9	Describe any efforts to address potential sources of bias	Methods (page 3-5)		
Study size	10	Explain how the study size was arrived at	Methods (page 3-5)		
Quantitative variables	11	Explain how quantitative variables were handled in the analyses. If applicable, describe which groupings were chosen, and why	Methods (page 3-5)		
Statistical methods	12	(a) Describe all statistical methods, including those used to control for confounding<br>(b) Describe any methods used to examine subgroups and interactions<br>(c) Explain how missing data were addressed<br>(d) *Cohort study* - If applicable, explain how loss to follow-up was addressed<br>*Case-control study* - If applicable, explain how matching of cases and controls was addressed<br>*Cross-sectional study* - If applicable, describe analytical methods taking account of sampling strategy<br>(e) Describe any sensitivity analyses	Methods (page 3-5)		
Data access and cleaning methods		..		RECORD 12.1: Authors should describe the extent to which the investigators had access to the database population used to create the study population.	Transparency (page 12) and Methods (page 3-5)

For peer review only

				RECORD 12.2: Authors should provide information on the data cleaning methods used in the study.	
Linkage		..		RECORD 12.3: State whether the study included person-level, institutional-level, or other data linkage across two or more databases. The methods of linkage and methods of linkage quality evaluation should be provided.	Methods (page 3-5)
**Results**					
Participants	13	(a) Report the numbers of individuals at each stage of the study (*e.g.*, numbers potentially eligible, examined for eligibility, confirmed eligible, included in the study, completing follow-up, and analysed)<br>(b) Give reasons for non-participation at each stage.<br>(c) Consider use of a flow diagram	Results (page 6)	RECORD 13.1: Describe in detail the selection of the persons included in the study (*i.e.,* study population selection) including filtering based on data quality, data availability and linkage. The selection of included persons can be described in the text and/or by means of the study flow diagram.	Methods (page 3-5)
Descriptive data	14	(a) Give characteristics of study participants (*e.g.*, demographic, clinical, social) and information on exposures and potential confounders<br>(b) Indicate the number of participants with missing data for each variable of interest<br>(c) *Cohort study* - summarise follow-up time (*e.g.*, average and total amount)	Results (page 6)		
Outcome data	15	*Cohort study* - Report numbers of outcome events or summary measures over time<br>*Case-control study* - Report numbers in each exposure	Results (page 7)		

		category, or summary measures of exposure<br>*Cross-sectional study* - Report numbers of outcome events or summary measures			
Main results	16	(a) Give unadjusted estimates and, if applicable, confounder-adjusted estimates and their precision (e.g., 95% confidence interval). Make clear which confounders were adjusted for and why they were included<br>(b) Report category boundaries when continuous variables were categorized<br>(c) If relevant, consider translating estimates of relative risk into absolute risk for a meaningful time period	Results (page 6-9)		
Other analyses	17	Report other analyses done—e.g., analyses of subgroups and interactions, and sensitivity analyses	Results (page 6-9)		
**Discussion**					
Key results	18	Summarise key results with reference to study objectives	Discussion (page 10-11)		
Limitations	19	Discuss limitations of the study, taking into account sources of potential bias or imprecision. Discuss both direction and magnitude of any potential bias	Discussion (page 10-11)	RECORD 19.1: Discuss the implications of using data that were not created or collected to answer the specific research question(s). Include discussion of misclassification bias, unmeasured confounding, missing data, and changing eligibility over time, as they pertain to the study being reported.	Discussion (page 10-11)
Interpretation	20	Give a cautious overall interpretation of results considering objectives,	Discussion (page 10-11)		

For peer review only

		limitations, multiplicity of analyses, results from similar studies, and other relevant evidence			
Generalisability	21	Discuss the generalisability (external validity) of the study results	Discussion (page 10-11)		
**Other Information**					
Funding	22	Give the source of funding and the role of the funders for the present study and, if applicable, for the original study on which the present article is based	Other information, at end of manuscript (page 12)		
Accessibility of protocol, raw data, and programming code		..		RECORD 22.1: Authors should provide information on how to access any supplemental information such as the study protocol, raw data, or programming code.	Methods (page 3-5)

*Reference: Benchimol EI, Smeeth L, Guttmann A, Harron K, Moher D, Petersen I, Sørensen HT, von Elm E, Langan SM, the RECORD Working Committee. The REporting of studies Conducted using Observational Routinely-collected health Data (RECORD) Statement. *PLoS Medicine* 2015; in press.

*Checklist is protected under Creative Commons Attribution (CC BY) license.

For peer review only
